# FAM177A1 disrupts SIRT3-SOD2 signaling to drive mitochondrial dysfunction-mediated VSMC phenotypic switching in vascular remodeling

**DOI:** 10.7150/ijbs.128409

**Published:** 2026-02-26

**Authors:** Ruiqi Mao, Yi Guo, Ling Jiang, Shichen Bu, Ming Chen, Minglu Liang, Xinyuan Gao, Yichen Wu, Wenjing Xu, Zilong Chen, Kai Huang, Xiaoguang Li, Cheng Wang

**Affiliations:** 1 Department of Cardiology, Union Hospital, Tongji Medical College, Huazhong University of Science and Technology, Wuhan, China.; 2 Hubei Key Laboratory of Metabolic Abnormalities and Vascular Aging, Huazhong University of Science and Technology, Wuhan, China.; 3 Hubei Clinical Research Center for Metabolic and Cardiovascular Disease, Huazhong University of Science and Technology, Wuhan, China.; 4 Cardiovascular Center, Liyuan Hospital, Tongji Medical College, Huazhong University of Science and Technology, Wuhan, China.; 5 Department of Geriatrics, Union Hospital, Tongji Medical College, Huazhong University of Science and Technology, Wuhan, China.; 6 Department of Cardiology, Xiamen Cardiovascular Hospital of Xiamen University, School of Medicine, Xiamen University, Xiamen, Fujian, China.; 7 Department of Cardiovascular Surgery, Union Hospital, Tongji Medical College, Huazhong University of Science and Technology, Wuhan, China.; 8 State Key Laboratory of Metabolism and Regulation in Complex Organisms, Taikang Center for Life and Medical Sciences, School of Basic Medical Sciences, Wuhan University, Wuhan, China.; 9 Department of Rheumatology, Union Hospital, Tongji Medical College, Huazhong University of Science and Technology, Wuhan, China.

## Abstract

**Aims:**

Vascular remodeling involves structural and functional vascular changes in response to injury, aging, and disease. A key pathological feature is vascular smooth muscle cells (VSMCs) phenotypic switching, which is accompanied by mitochondrial dysregulation. Metabolic reprogramming resembling the Warburg effect alongside mitochondrial oxidative damage collectively drive this pathological VSMC transdifferentiation. We hypothesized that targeting mitochondrial ROS could restore mitochondrial integrity and enhance oxidative phosphorylation (OXPHOS) to counteract both oxidative damage and metabolic reprogramming in cardiovascular diseases associated with vascular remodeling. We proposed that the uncharacterized membrane-associated protein FAM177A1 drives VSMC mitochondrial oxidative impairment and metabolic reprogramming, thereby promoting VSMC phenotypic switching and vascular dysfunction.

**Methods and Results:**

We modeled vascular remodeling using global *Fam177a1* knockout rats subjected to carotid balloon injury, VSMC-specific AAV-mediated *Fam177a1* knockdown in carotid artery ligation mice, and using *ApoE*^-/-^ mice fed a 12-week high-fat diet to induce atherosclerosis; in vitro VSMCs with platelet-derived growth factor-bb (PDGF-BB) stimulation further elucidated FAM177A1's role in phenotypic switching. FAM177A1 expression was significantly elevated in injured and atherosclerotic aortas, while its deficiency suppressed neointimal hyperplasia and atherosclerosis development. FAM177A1 deficiency upregulated mitochondrial functional genes, enhanced mtDNA biogenesis, reduced ROS accumulation, maintained redox homeostasis, and preserved mitochondrial membrane potential (ΔΨm). Moreover, FAM177A1 deficiency enhanced oxidative phosphorylation (OXPHOS) while reducing glycolytic flux, thereby improving bioenergetic efficiency and promoting a contractile phenotype. Molecular analysis revealed that FAM177A1 disrupted SIRT3-SOD2 binding, leading to elevated SOD2 K68 acetylation which decreased SOD2 activity and stability. Under pathological condition, this dysregulated cascade increased mitochondrial ROS, impaired mitochondrial function, thereby accelerating VSMC phenotypic switching.

**Conclusion:**

We identify FAM177A1 as a key mitochondrial regulator that drives VSMC switching through SIRT3-SOD2 axis disruption. Targeting FAM177A1 restores redox-metabolic homeostasis through scavenging ROS and improving OXPHOS, establishing it as a novel therapeutic target against vascular remodeling.

## Introduction

Cardiovascular diseases (CVD), primarily stroke and ischemic heart disease, are the leading causes of global disability and mortality^1^, ^2^. Furthermore, the worldwide burden of CVD, driven by multiple risk factors, significantly contributes to rising healthcare costs^3^, ^4^. Reducing CVD incidence, premature disability, and the resultant increase in public health spending is therefore a critical priority for national health systems and researchers. Chronic vascular pathologies—including atherosclerosis, post-angioplasty restenosis, aortic aneurysm, and hypertension—are consistently characterized by vascular remodeling^5^-^7^. Consequently, targeting vascular smooth muscle cell (VSMC) phenotypic switching represents a promising strategy for preventing and treating these remodeling-associated diseases^8^.

Beyond well-studied growth factors like transforming growth factor (TGF-β)^9^, fibroblast growth factor (FGF)^10^ and platelet-derived growth factor BB (PDGF-BB)^11^, reactive oxygen species (ROS) emerge as critical factor regulating VSMC phenotypic switching^12^. While basal ROS levels are essential for vascular homeostasis^13^, dysregulation of ROS-generating or scavenging enzymes induces oxidative stress, driving pathological VSMC transdifferentiation and proliferation^14^. Elevated ROS levels are associated with atherosclerosis, intimal injury, and diabetes. Furthermore, PDGF-BB or bradykinin stimulation further increases VSMC ROS^15^. Crucially, ROS promote VSMC proliferation, migration, and altered differentiation marker expression—core hallmarks of phenotypic switching^16^. ROS also facilitate PDGF-BB- or angiotensin II (Ang-II)-induced switching and vascular remodeling^17^. In atherosclerosis, ROS promote the synthetic VSMC phenotype via the ELK-1/SRF pathway^18^, while under inflammation, ROS activate NF-κB, upregulating osteopontin and suppressing myocardin-dependent contractile markers like SM22^19^. Thus, oxidative stress is fundamentally implicated in VSMC switching during vascular remodeling.

Mitochondria serve as a major ROS source yet are highly susceptible to oxidative damage, frequently becoming dysfunctional in CVD^20^. During VSMC switching, mitochondria shift metabolism from oxidative phosphorylation (OXPHOS) to glycolysis^21^. This Warburg-like adaptation supports the heightened bioenergetic and biosynthetic demands of the proliferative/migratory state, facilitating switching and remodeling^22^, ^23^. Enhancing OXPHOS to suppress this reprogramming is thus a promising therapeutic strategy^22^, ^24^. However, pathological mitochondrial ROS (mtROS) often compromise VSMC mitophagy and mitochondrial OXPHOS^25^. Consequently, metabolic reprogramming and mitochondrial dysfunction frequently coexist during switching. Superoxide dismutase 2 (SOD2), the primary mitochondrial superoxide scavenger^26^, constitutes a key regulatory node. Nevertheless, whether targeting SOD2 can effectively break the vicious cycle by scavenging mtROS, restoring OXPHOS, and preventing pathological VSMC switching remains a fundamental question.

FAM177A1 is an evolutionarily conserved (human to zebrafish) membrane-associated protein, primarily Golgi-localized^27^, ^28^. Emerging evidence has implicated FAM177A1 in various pathological conditions, including neurodevelopmental disorders^28^, inflammatory diseases^29^-^31^, and cancer^32^. Biallelic FAM177A1 variants cause a defined neurodevelopmental disorder featuring macrocephaly, global developmental delay, intellectual disability, seizures, behavioral abnormalities, hypotonia, and gait disturbances in five individuals across three families^28^. FAM177A1 deficiency dysregulates pathways involving apoptosis, inflammation, and proliferation suppression in human fibroblasts and zebrafish larvae^33^. Furthermore, FAM177A1 critically regulates innate immune responses, including TRAF6-NF-κB and Toll-like receptor 4 (TLR4) signaling^29^, ^30^. Reduced expression correlates with decreased primary biliary cholangitis incidence^31^. Within the Golgi, FAM177A1 interacts with VPS13B, influencing lipid trafficking and membrane dynamics^27^. While these findings existed linking FAM177A1 to neurodevelopmental disorders, macrophage-mediated inflammation, and potential associations with proliferation, no study has investigated the connection between FAM177A1 and vascular remodeling. It's function and related molecular mechanisms in VSMC switching remain undefined. In this study, we revealed that FAM177A1 is a critical regulator of VSMC phenotypic switching and promotes atherosclerosis and neointimal hyperplasia. Mechanistically, FAM177A1 was found to regulate the cellular fate of VSMCs by disrupting mitochondrial redox homeostasis. We serendipitously discovered its mitochondrial localization, revealing a key role for this pool in modulating the mitochondrial SIRT3-SOD2-mtROS axis and mitochondrial bioenergetics. Thus, targeting FAM177A1 may represent a promising therapeutic strategy for intervening in vascular remodeling.

## Methods

### Animal experiments, anesthesia and euthanasia

*Fam177a1* knockout rats were constructed by Wuhan Cyagen Biotechnology. 20bp (AAGAAAGATGTGCTGCCCAC) were deleted on exon 2 of *Fam177a1* gene. *ApoE*^-/-^ and C57BL/6J mice were purchased from Vital River Laboratories. Male Sprague-Dawley rats were purchased from Wuhan Shubeili biotechnology. For the mouse carotid artery ligation and rat carotid artery balloon injury models, anesthesia was induced with 100% O_2_ / 4% isoflurane and maintained throughout the procedure using 100% O₂ / 2% isoflurane. At the end of the experiments, all mice were euthanized under deep anesthesia with 100% O₂ / 5% isoflurane, followed by tissue collection. All mice and rats were housed in specific pathogen-free (SPF) facilities at Huazhong University of Science and Technology under a 12-hour light/dark cycle, with free access to food and water. All animal experiments were conducted with permission the Animal Experimentation Committee of Huazhong University of Science and Technology (IACUC No. 4635), in compliance with the Animals (Scientific Procedures) Act of 1986 (United Kingdom), Directive 2010/63/EU of the European Parliament on the protection of animals used for scientific purposes, and the NIH Guide for the Care and Use of Laboratory Animals.

### Establishment of mouse carotid artery ligation model

Eight weeks old male C57BL/6J mice were injected the AAV-EnSM22a-shRNA-*Fam177a1*. Carotid artery ligation after anesthesia as previously described^34^ were subjected after 3 weeks. Two weeks after surgery, carotid arteries were collected, serially sectioned, and stained.

### Establishment of rat ballon injury model

Male Sprague-Dawley rats (210-230 g) and 2.0-French catheter were used for the carotid artery injury model. The surgical procedure was performed as previously described^33^. The contralateral untreated carotid artery was used as control. Carotid arteries were harvested at predetermined experimental time points, followed by serial sectioning and staining. For 3-TYP treatments, rats were randomly grouped after ballon-catheter injury and the 3-TYP group rats were injected of 3-TYP (50mg/kg/d) intraperitoneally once a day.

### Establishment of atherosclerosis model

Eight-week-old *ApoE*^-/-^ mice were fed with high-fat diet (D12108C, Medicience, Jiangsu, China) for 12 weeks. Mice were sacrificed after anesthesia to collect the blood, heart and aortas. The collected blood was centrifuged after overnight standing, and the supernatant was used for blood lipid profiling. The intact aorta was stained with Oil Red O and photographed (Servicebio). The heart was cryosectioned, with serial sections of the aortic sinus processed for HE, Masson, and Oil Red O staining.

## Results

### FAM177A1 is upregulated in VSMC during phenotypic switching

**Figure 1:**
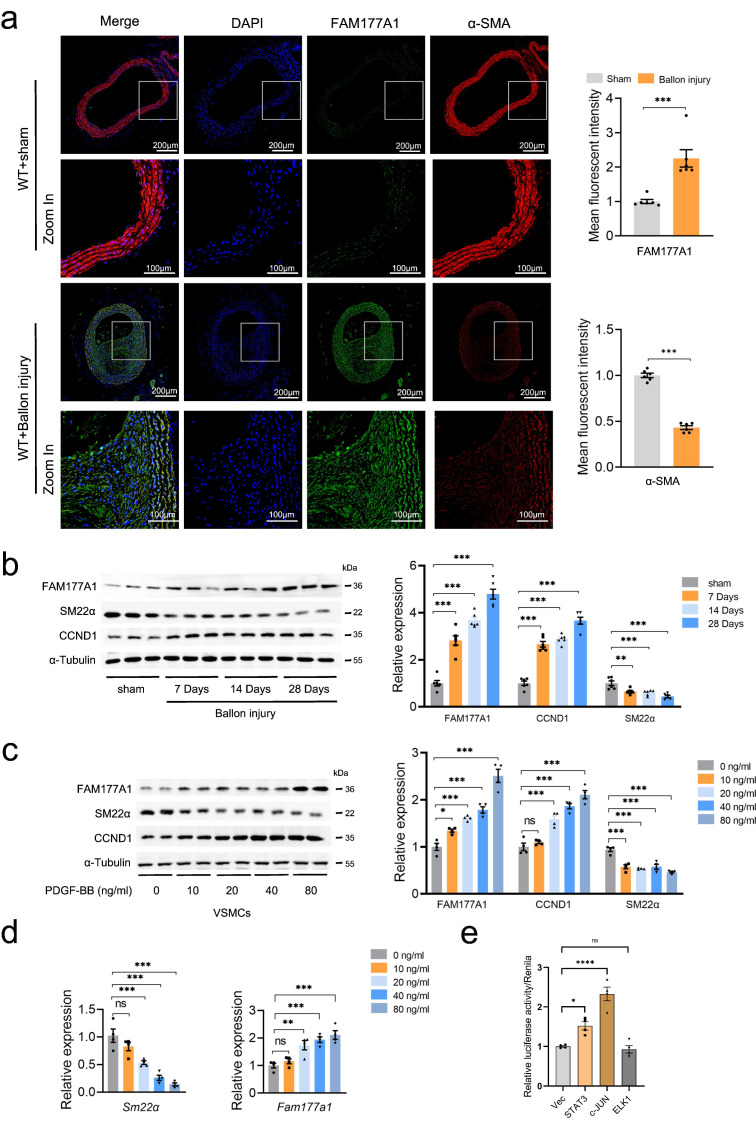
** FAM177A1 upregulation correlated with pathological vascular remodeling. a**. Representative immunofluorescence images and quantification of α-SMA (red) and FAM177A1 (green) in carotid arteries of WT+Ballon injury group (n = 6) and sham-operated group (WT+sham) (n = 6) post 28 days. Scale bar: 200 μm and 100 μm (zoom in). **b**. Representative Western blotting and quantification of FAM177A1, SM22α and CCND1 in the rat carotid arteries of sham-operated and post-ballon injury at different time intervals (n = 6). **c**. Representative Western blotting and quantification of CCND1, SM22α and Fam177a1 in rat VSMCs under different PDGF-BB concentrations (n = 3). **d**. qPCR analysis of the relative mRNA level of *Sm22α* and *Fam177a1* in rat VSMCs under different concentrate PDGF-BB stimulation (n = 3). 18S was used for control. **e**. Dual-luciferase reporter assays performed to measure *Fam177a1* promoter activity in A7r5 cells transfected with empty vector (Vec) or STAT3, c-JUN, and ELK1 overexpression plasmids for 48 hours (n = 3). Vec was used for control. All Data are presented as means ± SEM. P-values are calculated using the Student's t-test (unpaired) in **a** and one-way ANOVA with a post hoc test of Tukey's analysis in **b-e**,. ns, *p*>0.05, *p <0.05, **p<0.01, ***p<0.001. SM22α, smooth muscle 22 alpha; CCND1, cyclin D1; PDGF-BB, platelet-derived growth factor-bb.

To investigate the connection between FAM177A1 and VSMCs, published single-cell RNA sequencing (scRNA-seq) data (GSE174098) derived from left (control) and right (balloon injury) carotid arteries of rats were applied to analyze *Fam177a1* dynamic in vascular injury stenosis. A t-SNE dimension reduction plot of 7 cell types showed the proportions of different cell types, including endothelial cells (ECs), VSMCs, fibroblasts (FB), macrophages and unknown cells (Fig. S1a). Through vascular remodeling, VSMC phenotype switched to fibroblast phenotype, accompanied by loss of contractile VSMCs markers and elevation of cell proliferation, migration and ECM remodeling markers^35^, ^36^. Supportingly, levels of SMC-specific marker genes (*Acta2, Tagln, Cnn1*) were declined in FB cell group (Fig. S1b-c). Noteworthily, the *Fam177a1* expression was observed tendency towards higher in FB compared to VSMC (Fig. S1c). We next examined the expression of FAM177A1 by constructing rat carotid balloon injury. Rats were sacrificed 0/7/14/28 days after surgery and carotid artery specimens were collected from the sham group (Sham) and the surgical group (Injury). Immunofluorescence assay showed that FAM177A1 was significantly upregulated in the hyperplastic intima of rat carotid arteries 28 days after balloon injury, accompanied with decreased α-SMA (VSMCs identity marker) (Fig. 1a). Moreover, western blots showed time-dependent upregulation of FAM177A1 expression with a concomitant reduction in SM22α (contractile marker) and elevation in cyclin D1 (CCND1, proliferation marker) in extraction of carotid artery from carotid ballon injury rats, compared with sham group (Fig. 1b). Next, we performed *in vitro* experiments that quiescent-state primary VSMCs were stimulated with PDGF-BB to induce VSMC phenotypic switching^37^, ^38^. Consistently, we observed PDGF-BB concentration dependent dynamic of decreased SM22α and increased CCND1 (Fig. 1c), indicating a contractile quiescent phenotype switching to a synthetic one. Importantly, the protein expression of FAM177A1 was increasing upon the increased PDGF-BB stimulation (Fig. 1c). Along this vein, we found that *Fam177a1* mRNA levels were significantly elevated in a PDGF-BB concentration-dependent manner as well (Fig. 1d). Based on the observation that FAM177A1 expression changed at the RNA level following PDGF-BB stimulation, we further employed luciferase reporter assays to investigate which specific transcription factors or signaling pathways control its expression under PDGF-BB stimulation. Transfection with STAT3 or c-JUN plasmids promoted the luciferase activity/renilla ratio, thereby enhancing FAM177A1 transcription, whereas transfection with ELK1 plasmid did not significantly promote transcription. Collectively, these data suggested that both c-Jun/AP-1 and STAT3 are potential transcription factors mediating the PDGF-BB-induced transcriptional upregulation of FAM177A1(Fig. 1e).

Additionally, we established a conventional atherosclerotic (AS) model using *ApoE*^-/-^ mice induced with a 12-week high-fat diet (HFD). We observed that FAM177A1 expression was upregulated in VSMCs within atherosclerotic plaques, concomitant with a decline in α-SMA expression (Fig. S1d). These findings suggest that FAM177A1 is upregulated during HFD-induced VSMC phenotypic transformation in mice. Humans express two FAM177A1 isoforms (isoform 1 and 2), resulting from alternative initiation sites that generate distinct N-terminal regions due to the exclusion or inclusion of a 21-amino acid fragment^39^. Notably, humans also produce a secreted form containing an N-terminal 21-amino acid signal peptide (the signal peptide was predicted by SignalP6.0), detectable in human blood^40^. To investigate the clinical relevance of FAM177A1 in AS, we collected serum samples and clinical records from 99 individuals undergoing routine health examinations (Table S2). Based on coronary angiography (stenosis ≥50%) and carotid ultrasound (presence of plaque or intima-media thickness ≥1.0 mm) criteria, 45 subjects (37 male, 8 female) were categorized into the AS group. The remaining 54 subjects (44 male, 10 female) served as healthy controls. ELISA analysis revealed significantly elevated serum FAM177A1 levels in AS patients (189.2 ± 33.47 pg/ml) compared to controls (167.6 ± 35.54 pg/ml; p = 0.0025) (Fig. S1e). Its elevated serum levels in AS patients demonstrate considerable translational potential as a diagnostic biomarker for atherosclerosis.

In summary, these findings strongly suggested that FAM177A1 is upregulated in VSMC during phenotypic switching, and suggested that it may play a crucial role in vascular remodeling and maintaining the contractile phenotype of VSMCs.

### *Fam177a1* knockout suppresses vascular remodeling in balloon injury-induced neointimal hyperplasia and atherosclerosis

**Figure 2:**
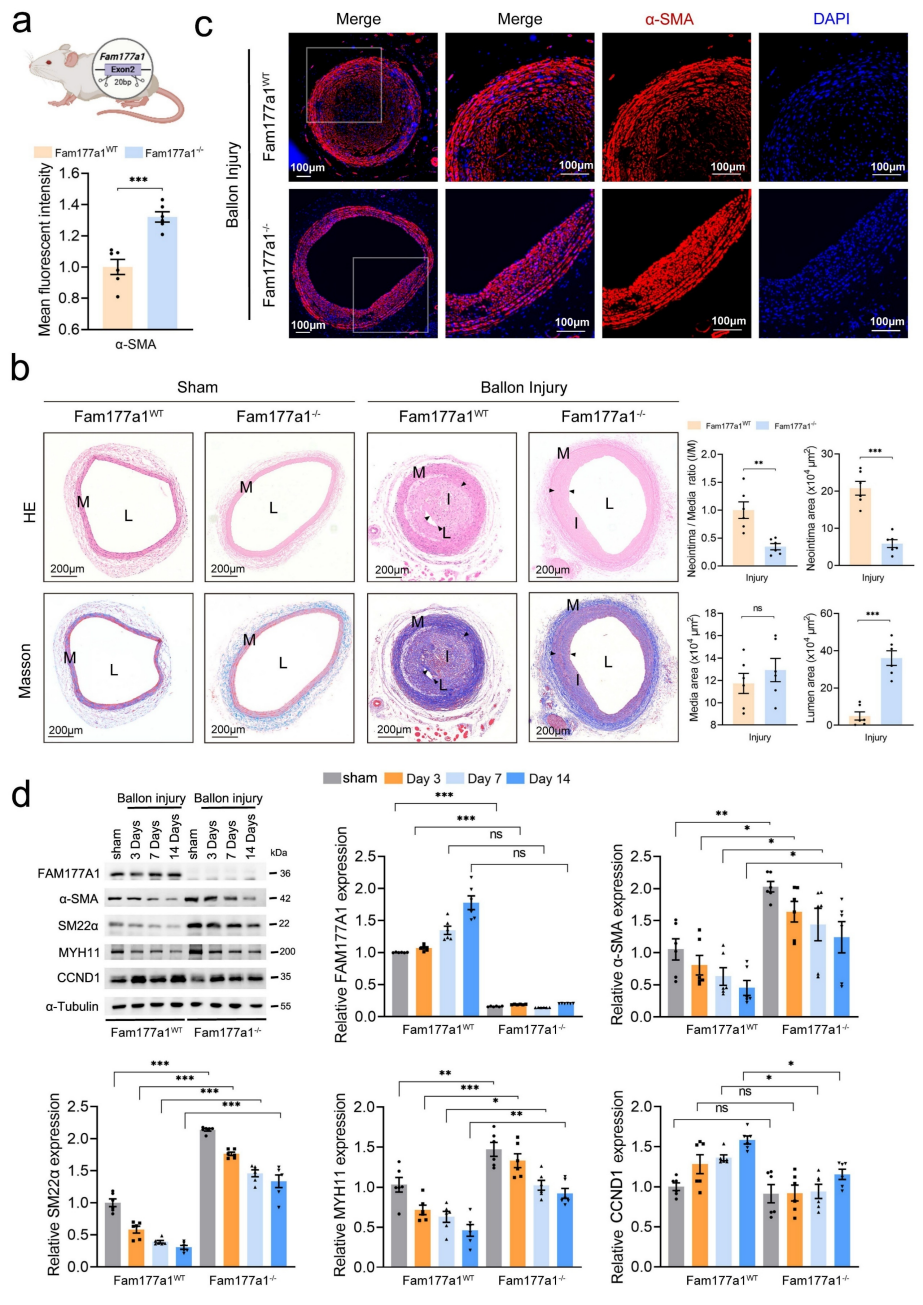
***Fam177a1* knockout alleviated vascular intimal hyperplasia *in vivo*. a.** Schematic illustration of the generation of *Fam177a1* knockout rat. **b**. Representative immunofluorescence images and quantification of α-SMA (red) in male Fam177a1^WT^ and Fam177a1^-/-^ rats carotid arteries post ballon injury 28 days (n = 6). DAPI was used for nuclear staining (blue). Scale bar: 100μm. Statistical analysis of αSMA mean fluorescence intensity (total intensity/area) were shown on the left (normalized to Fam177a1^WT^ group). **c**. **Left**, Representative cross sections of H&E and Masson in male Fam177a1^WT^ and Fam177a1^-/-^ rats carotid arteries post ballon injury 28 days. Scale bar=200μm. **Right**, quantitative analysis of the neointima/media ratio(I/M), neointima areas, media areas and lumen areas in HE-stained sections from male *Fam177a1*^WT^ and *Fam177a1*^-/-^ rats carotid arteries post ballon injury 28 days (n = 6). **d.** Representative Western blotting and quantification in the *Fam177a1*^WT^ and *Fam177a1*^-/-^ rats carotid arteries of sham-operated or post balloon injury at different time intervals groups (n = 6). α-Tubulin was used for control. Data were presented as relative fold change to *Fam177a1*^WT^ sham group. All Data are presented as means ± SEM. P-values are calculated using the Student's t-test (unpaired) in **b** and** c**, and one-way ANOVA with a post hoc test of Tukey's analysis in **d**. *p <0.05, **p<0.01, ***p<0.001. H&E, Hematoxylin and Eosin; α-SMA, α smooth muscle actin**;** MYH11, myosin heavy chain 11.

To further elucidate the function of FAM177A1 in vascular remodeling, we generated *Fam177a1* knockout (*Fam177a1*^-/-^) rats through a 20-bp deletion in exon 2 of the gene (Fig. 2a). Next, we used 1.5mm balloon catheter to construct the carotid artery neointima injury model on *Fam177a1*^-/-^ and WT (*Fam177a1*^WT^) rats. H&E and Masson staining revealed significant compensatory dilatation in these intact arteries, which likely developed in response to luminal stenosis caused by neointimal hyperplasia in the operated vessels (Fig. 2b). Moreover, genetic ablation of *Fam177a1* suppressed postinjury neointima formation (20.79±4.536 x10^4^μm^2^ vs 5.88±2.581 x10^4^μm^2^, p<0.0001) and collagen deposition (Fig. 2b). Compared to *Fam177a1*^WT^ rat, *Fam177a1*^-/-^ rat exhibited a significantly larger lumen area (4.963±5.387 x10^4^μm^2^ vs 36.08±9.552 x10^4^μm^2^, p<0.0001) (Fig. 2b). These data indicated that *Fam177a1* knockout suppresses vascular remodeling in balloon injury-induced neointimal hyperplasia. Additionally, immunofluorescence assays revealed that carotid arteries from *Fam177a1*^-/-^ rat exhibited higher levels of α-SMA protein compared with those from *Fam177a1*^WT^ rat following injury (Fig. 2c), indicating enhanced contractile function. Western blots analysis of injured vessels demonstrated that FAM177A1 deficiency upregulated VSMC contractile proteins (α-SMA, SM22α, MYH11) and downregulated the proliferation marker CCND1 at sequential time points post-injury (0, 3, 7, and 14 days) (Fig. 2d).

In parallel, we constructed recombinant AAV9 vectors carrying either a short hairpin RNA targeting mouse *Fam177a1* (AAV-EnSM22α-sh*Fam177a1*, targeting sequence: GCAGAUAAGUAAUGAAAGA) or a scramble shRNA control (AAV-EnSM22α-shScr), both driven by the vascular smooth muscle-specific SM22α promoter. Three weeks after tail vein injection of these recombinant AAVs, western blot analysis confirmed the successful knockdown of FAM177A1 in the aorta (Fig. S2a). Next, we utilized these recombinant AAVs in a conventional mouse carotid artery injury model in which the left common carotid artery was ligated with a 6-0 silk suture (Fig. S2b). Consistent with our previous results, H&E staining revealed that wire injury-induced neointima formation was attenuated in the carotid arteries of mice injected with AAV-SM22α-sh*Fam177a1* (Fig. S2c). These findings demonstrate a prominent regulatory role for FAM177A1 in neointima formation following carotid artery ligation.

**Figure 3:**
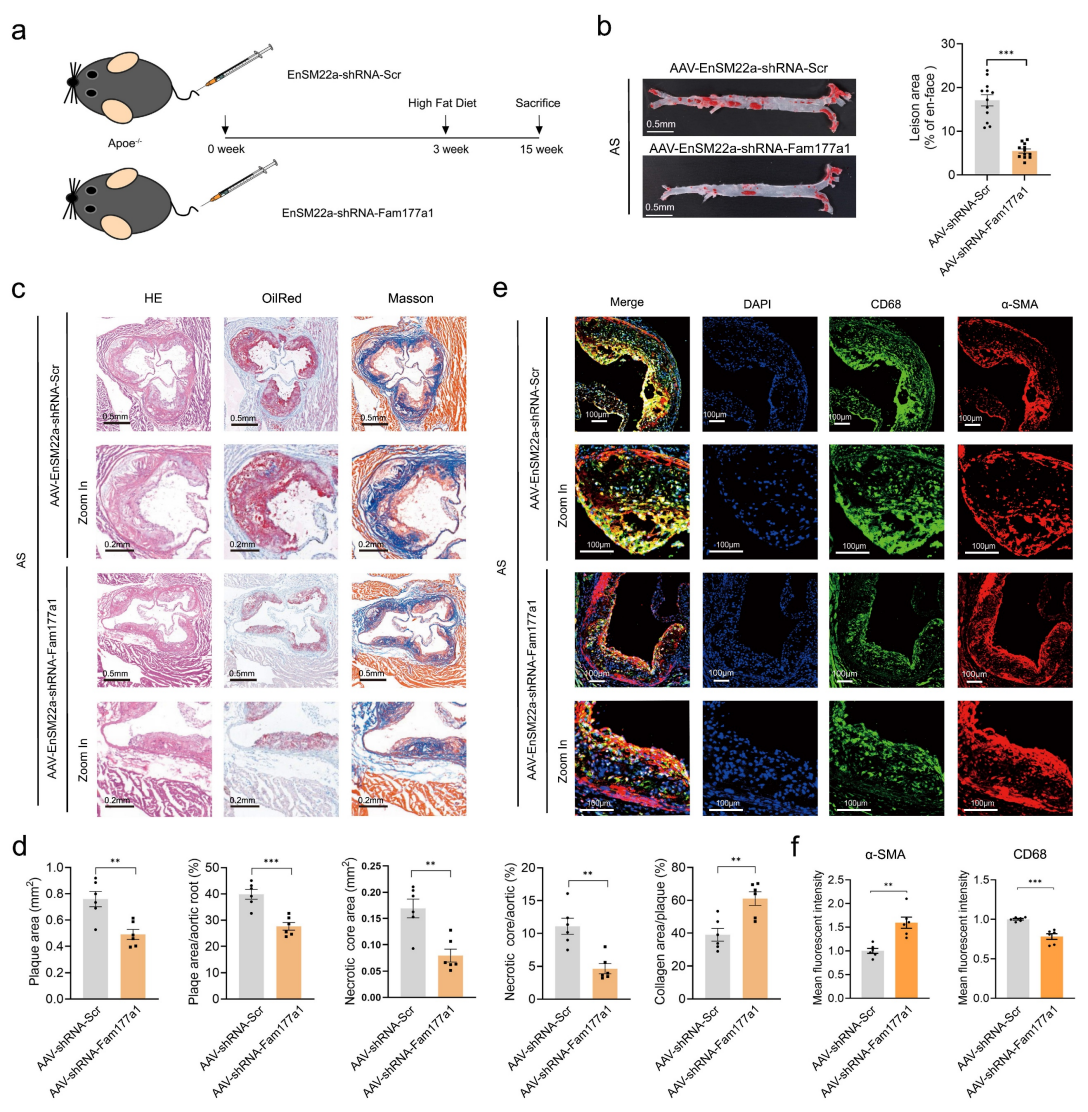
***Fam177a1* specific knockdown in VSMCs mitigated atherosclerosis *in vivo*. a.** Experimental scheme. *ApoE*^-/-^ mice were injected with AAV-EnSM22α-shRNA-Scramble or AAV-EnSM22α-shRNA-*Fam177a1* via the tail vein, followed by a 12-week high-fat diet to establish the atherosclerosis model with smooth muscle-specific knockout of *Fam177a1*. Blood, aortas, and heart were collected for further analyses. **b**. Representative image of Oil Red O-stained whole aortas from mice as indicated (n= 12). The relative En face atherosclerotic plaque area was quantified compared to AAV-EnSM22α-shRNA-Scramble group. Scale bar: 0.5mm.** c**. Representative image of cross section of the H&E, Oil red O and Masson-stained aortic roots from mice as indicated. Scale bar: 0.5mm and 0.2mm (zoom in).** d**. Quantitative analysis of plaque areas, plaque/aortic area ratio and necrotic areas in H&E, Oil red O and Masson-stained aortic roots from mice as indicated (n = 6). Data were compared to AAV-EnSM22α-shRNA-Scramble group. **e**. Representative immunofluorescence images of CD68(green) and αSMA (red) on cross section of the aortic roots of indicated mice (n = 6). DAPI was used for nuclear staining(blue). Scale bar: 100μm. **f**. Statistical analysis of CD68 and αSMA mean fluorescence intensity. All Data are presented as means ± SEM. P-values are calculated using the Student's t-test (unpaired) in **b, d** and** f**. *p <0.05, **p<0.01, ***p<0.001. Scr, Scramble.

To investigate the function of FAM177A1 in atherosclerosis-associated vascular remodeling, *ApoE*^-/-^ mice fed with HFD for 12 weeks were injected with either AAV-EnSM22α-sh*Fam177a1* or AAV-EnSM22α-Scr (Fig. 3a). No significant differences were observed in liver Oil Red O staining or blood lipid levels between the sh*Fam177a1* and shScr groups (Fig. S2d-e), indicating comparable systemic metabolic profiles. En-face Oil Red O staining of the entire aorta revealed that *Fam177a1* knockdown in VSMCs via AAV-mediated SM22α promoter-driven shRNA delivery significantly reduced plaque area (Fig. 3b). Analysis of Oil Red O-stained aortic roots further demonstrated reduced plaque area in the sh*Fam177a1* group (0.4899 ± 0.09239 mm² vs. 0.7590 ± 0.1433 mm², p=0.0031) (Fig. 3c, d). Furthermore, H&E and Masson staining of aortic roots showed that *Fam177a1* knockdown resulted in a smaller necrotic core area (0.07944 ± 0.03022 mm² vs. 0.1691 ± 0.04373 mm², p=0.0020) and altered collagen content within the lesions (Fig. 3c, d). These findings suggest that *Fam177a1* knockdown reduces atherosclerotic plaque burden and promotes plaque stability.

Given that plaque rupture is inversely correlated with contractile VSMC abundance and positively correlated with VSMC-derived macrophage-like cells^41^-^44^, we assessed VSMC and macrophage markers via immunofluorescence. The aortic root lesions of the AAV-SM22α-sh*Fam177a1* group exhibited higher levels of α-SMA (VSMCs marker) and lower levels of CD68 (macrophage marker) compared to controls (Fig. 3e, f). Collectively, these results indicate that FAM177A1 regulates atherosclerosis progression and plaque stability, potentially by modulating VSMC phenotype and function.

### FAM177A1 promotes VSMC phenotype switching from the contractile to synthetic phenotype

Given that FAM177A1 play a vital role in atherosclerosis and neointimal hyperplasia in in the tunica media, we next conducted in vitro cellular experiments to investigate the effect of FAM177A1 on VSMC phenotype. At quiescent state without PDGF-BB stimulation, immunofluorescence staining of F-actin revealed that *Fam177a1* knockout preserved the spindle-like morphology of rat primary VSMCs, preventing a shift toward large, polygonal shapes observed in controls (Fig.S3a). Conversely, FAM177A1 overexpression induced a marked transition from spindle-shaped to polygonal morphology (Fig.S3d), indicating a synthetic phenotype. F*am177a1*^-/-^ VSMCs exhibited upregulated contractile proteins (α-SMA, MYH11, SM22α, CNN1) and downregulated proliferation markers (PCNA, CCND1) at both mRNA and protein levels (Fig.S3b, c). In contrast, FAM177A1-overexpressing VSMCs showed attenuated contractile protein expression and elevated proliferation markers (Fig.S3e, f). These results demonstrate that FAM177A1 promotes cell-autonomous VSMC phenotype switching.

### FAM177A1 exacerbates PDGF-BB-induced VSMC phenotypic switching

**Figure 4:**
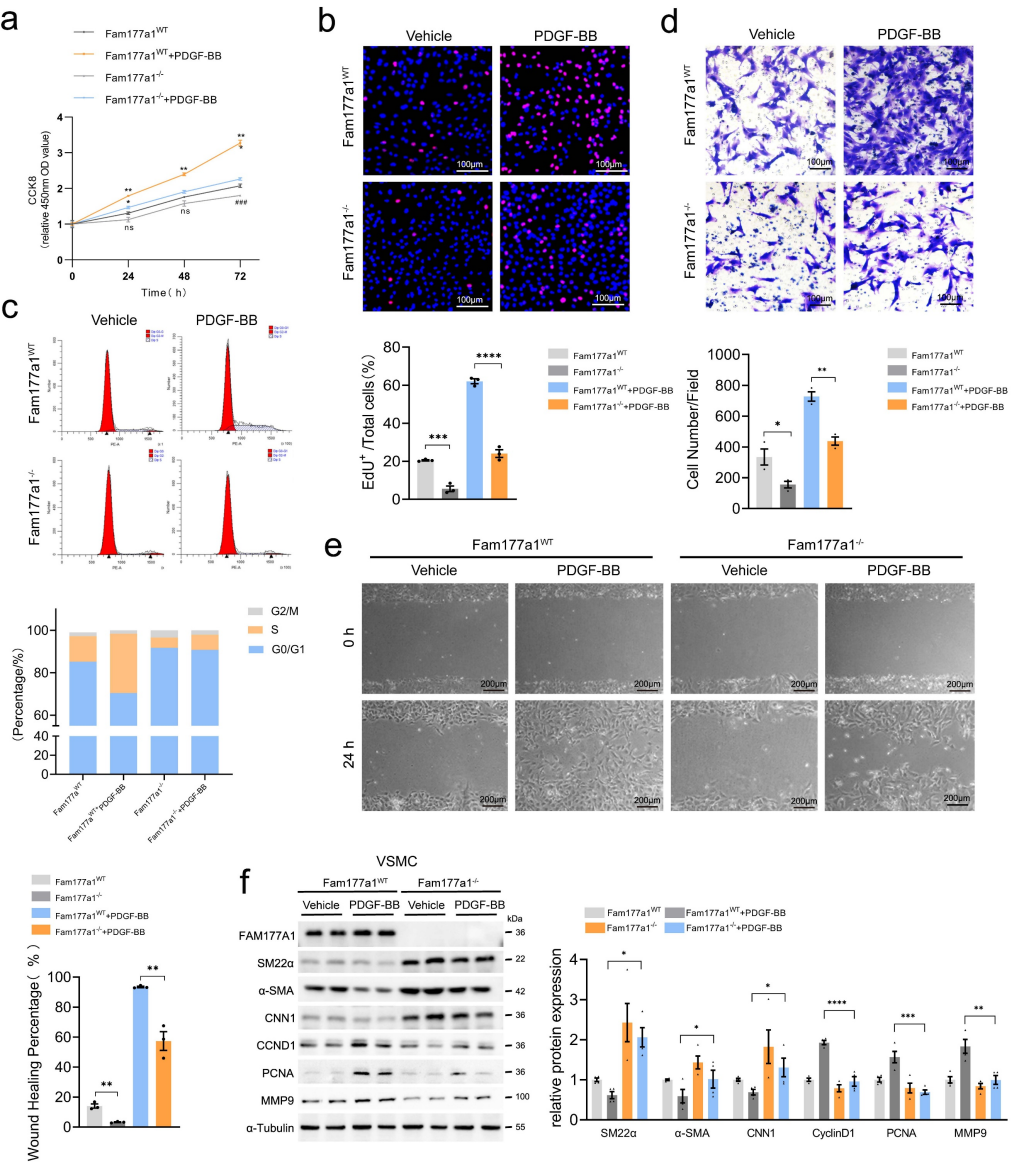
***Fam177a1* knockout preserved VSMCs contractile phenotype *in vitro*.** *Fam177a1*^WT^ and *Fam177a1*^-/-^ rat VSMCs were stimulated with vehicle or PDGF-BB (20ng/ml). Cell proliferation was examined by CCK8 assays (**a**), EdU staining (**b**) and cell cycle flow cytometry (**c**) (**b**, scale bar: 100μm; **d**, scale bar: 100μm; **e**, scale bar: 200μm). Cell migration was examined using Transwell (**d**) and wound healing assays (**e**). **f**. Representative Western blotting and quantification of FAM177A1, SM22α, αSMA, CNN1, PCNA, MMP9 expression in *Fam177a1*^WT^ and *Fam177a1*^-/-^ rat VSMCs after PDGF-BB (20ng/ml, 48h) induction. All Data are presented as means ± SEM. P-values are calculated using the one-way ANOVA with a post hoc test of Tukey's analysis in **a-f**. *p <0.05, **p<0.01, ***p<0.001. CNN1, calponin-1; PCNA, proliferating cell nuclear antigen; MMP9, matrix metalloproteinase-9.

To further explore the regulation of VSMC phenotype by FAM177A1 under pathological conditions, we treated VSMCs with PDGF-BB (20 ng/ml) to simulate a disease-relevant microenvironment. Under PDGF-BB stimulation mimicking pathological conditions, *Fam177a1* knockout significantly decreased CCK-8 activity, EdU^+^ cell proportion, and S-phase fraction in primary VSMCs compared to *Fam177a1*^WT^ (Fig. 4a-c), indicating suppressed proliferation. Transwell and wound healing assays confirmed that *Fam177a1* knockout inhibited PDGF-BB-induced VSMC migration (Fig. 4d-e). Consistent with this, *Fam177a1*^-/-^ VSMCs displayed elevated contractile proteins (SM22α, α-SMA, CNN1), and suppressed proliferation (Cyclin D1, PCNA) and migration markers (MMP9) (Fig. 4f). Conversely, FAM177A1 overexpression exacerbated PDGF-BB-induced VSMC proliferation, migration, and contractile-synthetic protein shifts (Fig.S4a-i).

Collectively, these results establish FAM177A1 as a novel modulator of VSMC phenotypic plasticity, governing the contractile-synthetic transition. *Fam177a1* knockout blocked PDGF-BB-mediated transdifferentiation by sustaining contractile programs while suppressing proliferation/migration, whereas overexpression accelerated switching toward a synthetic state.

### FAM177A1 regulates VSMC mitochondrial oxidative phosphorylation and function

**Figure 5:**
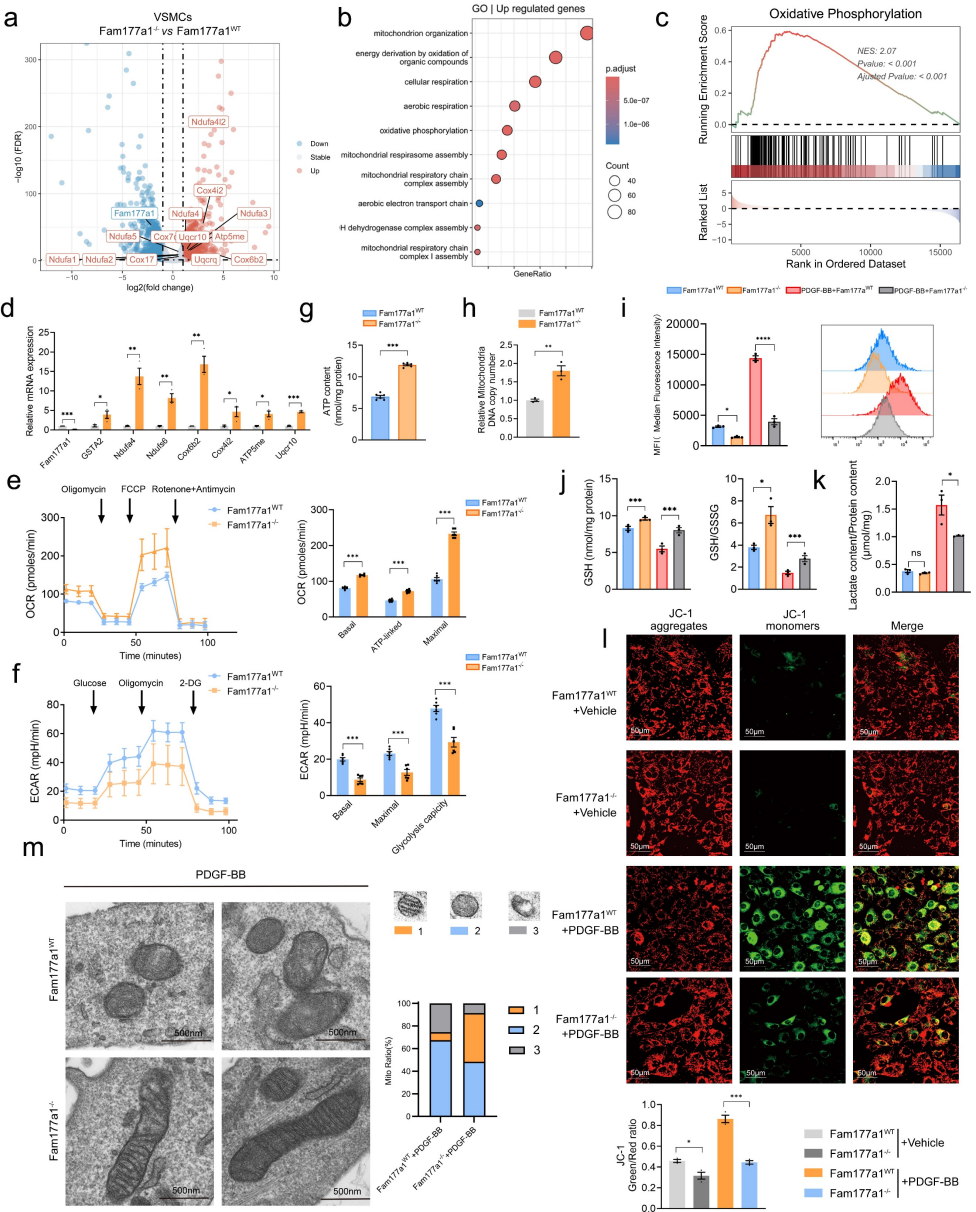
***Fam177a1* knockout exhibited mitochondrial oxidative phosphorylation protective function of VSMCs. a.** Volcano plot displaying differentially expressed genes (DEGs) of interest in *Fam177a1*^WT^ and *Fam177a1*^-/-^ rat VSMCs through transcriptome sequencing (n = 3). **b**. Mitochondria function related GO enrichment analysis of up-regulated genes in *Fam177a1*^-/-^ VSMCs. **c-d**. GSEA enrichment analysis of RNA-seq data (**c**) and qPCR validation of mitochondria and oxidative phosphorylation related DEGs (**d**) (n = 3). 18S was used for control. Data were presented as relative fold change to *Fam177a1*^WT^ VSMCs. **e**. Mitochondria oxygen consumption rate (OCR) of *Fam177a1*^WT^ and* Fam177a1*^-/-^ VSMCs measured using Seahorse XF24 flux analyzer. Mitochondrial effectors were added at the indicated time points (arrows, n = 3).** f**. Extracellular acidification rate (ECAR) of *Fam177a1*^WT^ and *Fam177a1*^-/-^ VSMCs measured using Seahorse XF24 flux analyzer, with sequential injections at time points (arrows, n = 3). **g**. Intracellular ATP production content in *Fam177a1*^WT^ and *Fam177a1*^-/-^ VSMCs (n = 3). **h**. qPCR of mtDNA copy number in VSMCs as indicated (n = 3). **i**. Flow cytometry analysis of ROS production in *Fam177a1*^WT^ and *Fam177a1*^-/-^ VSMCs after PDGF-BB (20ng/ml, 48h) induction (n = 3). **j**. GSH content and GSH/GSSG ratio in *Fam177a1*^WT^ and *Fam177a1*^-/-^ VSMCs after PDGF-BB (20ng/ml, 48h) induction (n = 3). **k**. Lactate content/ Protein ratio in *Fam177a1*^WT^ and *Fam177a1*^-/-^ VSMCs after PDGF-BB (20ng/ml, 48h) induction (n = 3). **l**. Representative immunofluorescence images and quantification of JC-1 staining showing membrane potential by confocal fluorescence microscopy of *Fam177a1*^WT^ and *Fam177a1*^-/-^ VSMCs after PDGF-BB (20ng/ml, 48h) induction (n = 3) (scale bar: 50 μm). **m**. Representative electron micrographs of mitochondria in *Fam177a1*^WT^ and *Fam177a1*^-/-^ VSMCs following PDGF-BB (20ng/ml, 48h) stimulation (scale bar: 500nm). Mitochondria were classified into three functional categories based on morphological vacuolization and cristae abundance. The proportional distribution of each mitochondrial morphology was shown in the bar graph. All Data are presented as means ± SEM. P-values are calculated using the Student's t-test (unpaired) in **d**-**g** and **l** and one-way ANOVA with a post hoc test of Tukey's analysis in data **h**-**k**. *p <0.05, **p<0.01, ***p<0.001. GSTA2, glutathione S-transferase alpha 2; Ndufa4, NADH dehydrogenase (ubiquinone) 1 alpha subcomplex subunit 4; Ndufs6, NADH dehydrogenase (ubiquinone) iron-sulfur protein 6; Cox6b2, Cytochrome c oxidase subunit 6B2; Cox4i2, Cytochrome c oxidase subunit 4 isoform 2; ATP5me, ATP synthase membrane subunit e; Uqcr10, Ubiquinol-cytochrome c reductase complex subunit 10; OCR, oxygen consumption rate; ECAR, extracellular acidification rate.

After establishing FAM177A1's role in orchestrating VSMC phenotypic switching both *in vitro* and* in vivo*, the underlying cellular mechanisms remained unknown. To characterize its function in VSMCs, we performed bulk mRNA sequencing on *Fam177a1*^WT^ and *Fam177a1*^-/-^ rat primary VSMCs. This analysis identified 1007 upregulated genes and 870 downregulated genes in *Fam177a1*^-/-^ VSMCs (Fig. 5a). Gene Ontology (GO) and Gene Set Enrichment Analysis (GSEA) of differentially expressed genes revealed significant enrichment in mitochondrial pathways, particularly oxidative phosphorylation (Fig. 5b, c). Suppressed oxidative phosphorylation and enhanced glycolysis in VSMCs, is a recognized driver of phenotypic switching^45^, ^46^. We therefore hypothesized that FAM177A1 modulates phenotypic switching through regulation of VSMC mitochondrial function. Quantitative PCR assays confirmed expression changes in DEGs involved in oxidative phosphorylation and mitochondrial respiratory chain complexes, including *GSTA2, Ndufa4, Ndufs6, Cox6b2, Cox4i2, ATP5me,* and* Uqcr10* in *Fam177a1*^-/-^ VSMCs (Fig. 5d). To functionally assess mitochondrial respiration, we measured oxygen consumption rate (OCR) using Seahorse XF24 flux analysis. Sequential administration of inhibitors showed enhanced basal and maximal mitochondrial respiration in *Fam177a1*^-/-^ VSMCs (Fig. 5e). The ATP synthase inhibitor oligomycin induced a more pronounced OCR reduction in knockout cells, indicating greater ATP-linked respiration (Fig. 5e). Furthermore, *Fam177a1*^-/-^ VSMCs exhibited decreased extracellular acidification rate (ECAR) (Fig. 5f), reflecting suppressed glycolytic activity. ATP quantification assays confirmed significantly higher ATP production in *Fam177a1*^-/-^ compared to *Fam177a1*^WT^ controls (Fig. 5g). These collective findings demonstrate that FAM177A1 deficiency enhances mitochondrial oxidative phosphorylation while reducing glycolytic flux, thereby promoting a contractile VSMC phenotype through improved bioenergetic efficiency.

### FAM177A1 deficiency mitigates PDGF-BB-induced mitochondrial dysfunction

To further elucidate the underlying mechanism of FAM177A1 regulating PDGF-BB-induced mitochondrial dysfunction, we performed in vitro assays to measure ROS levels, glutathione (GSH/GSSG) ratio, mitochondrial DNA (mtDNA) copy number, mitochondrial membrane potential and lactate production upon PDGF-BB stimulation. Under pathological conditions such as vascular injury, atherosclerosis, calcification, and senescence—all closely linked to mitochondrial dysfunction—vascular disease-related mitochondrial impairment during VSMC phenotypic switching accelerates disease progression^47^, ^48^. This impairment is characterized by suppressed oxidative phosphorylation, enhanced glycolysis, and accumulated reactive oxygen species (ROS)^49^, ^50^. Such mitochondrial dysfunction critically underscores the importance of mitochondrial homeostasis for restoring VSMC contractile function.

As anticipated, flow cytometry detected markedly increased ROS levels during PDGF-BB-induced phenotypic switching (Fig. 5i), indicating mitochondrial oxidative stress. Importantly, *Fam177a1* knockout effectively attenuated PDGF-BB-stimulated ROS production (Fig. 5i). Assessment of cellular redox status via GSH/GSSG measurements revealed elevated GSH content and increased GSH/GSSG ratios in *Fam177a1*^-/-^ VSMCs (Fig. 5j), demonstrating maintained cellular reduction capacity and protection against PDGF-BB-induced oxidative stress. Lactate content results showed that while lactate levels were slightly lower in the *Fam177a1*^-/-^ VSMCs group compared to the *Fam177a1^WT^* group, this difference was not statistically significant. However, upon PDGF-BB stimulation, lactate production was significantly suppressed in the *Fam177a1*^-/-^ VSMCs group. This observation aligns with the finding that *Fam177a1*^-/-^ VSMCs inhibits glycolytic flux and provides further evidence that *Fam177a1*^-/-^ VSMCs can suppress the glycolytic pathway, stabilize mitochondrial function, and reduce the generation of metabolic byproducts from glycolysis (Fig. 5k). Impaired oxidative phosphorylation leads to mitochondrial damage, including dissipated mitochondrial membrane potential (ΔΨm), reduced mtDNA copy number, and morphological abnormalities. Using JC-1 staining, we observed that PDGF-BB stimulation significantly dissipated ΔΨm (reduced red/green fluorescence ratio). This effect was reversed in *Fam177a1*^-/-^ VSMCs, which maintained preserved ΔΨm (Fig. 5l). Additionally, *Fam177a1*^-/-^ VSMCs exhibited significantly increased mtDNA content (Fig. 5h). Electron microscopy analysis also revealed that *Fam177a1*^-/-^ VSMCs exhibited a higher proportion of mitochondria with intact and well-defined cristae, whereas the percentage of mitochondria containing obscured/disintegrated cristae or vacuolar structures was significantly reduced (Fig. 5m).

Collectively, these findings demonstrate that FAM177A1 deficiency enhances mitochondrial oxidative phosphorylation, upregulates mitochondrial functional genes, reduces ROS generation, maintains redox homeostasis, preserves ΔΨm, increases mtDNA biogenesis, and mitigates mitochondrial dysfunction.

### FAM177A1 interacts with SOD2

**Figure 6:**
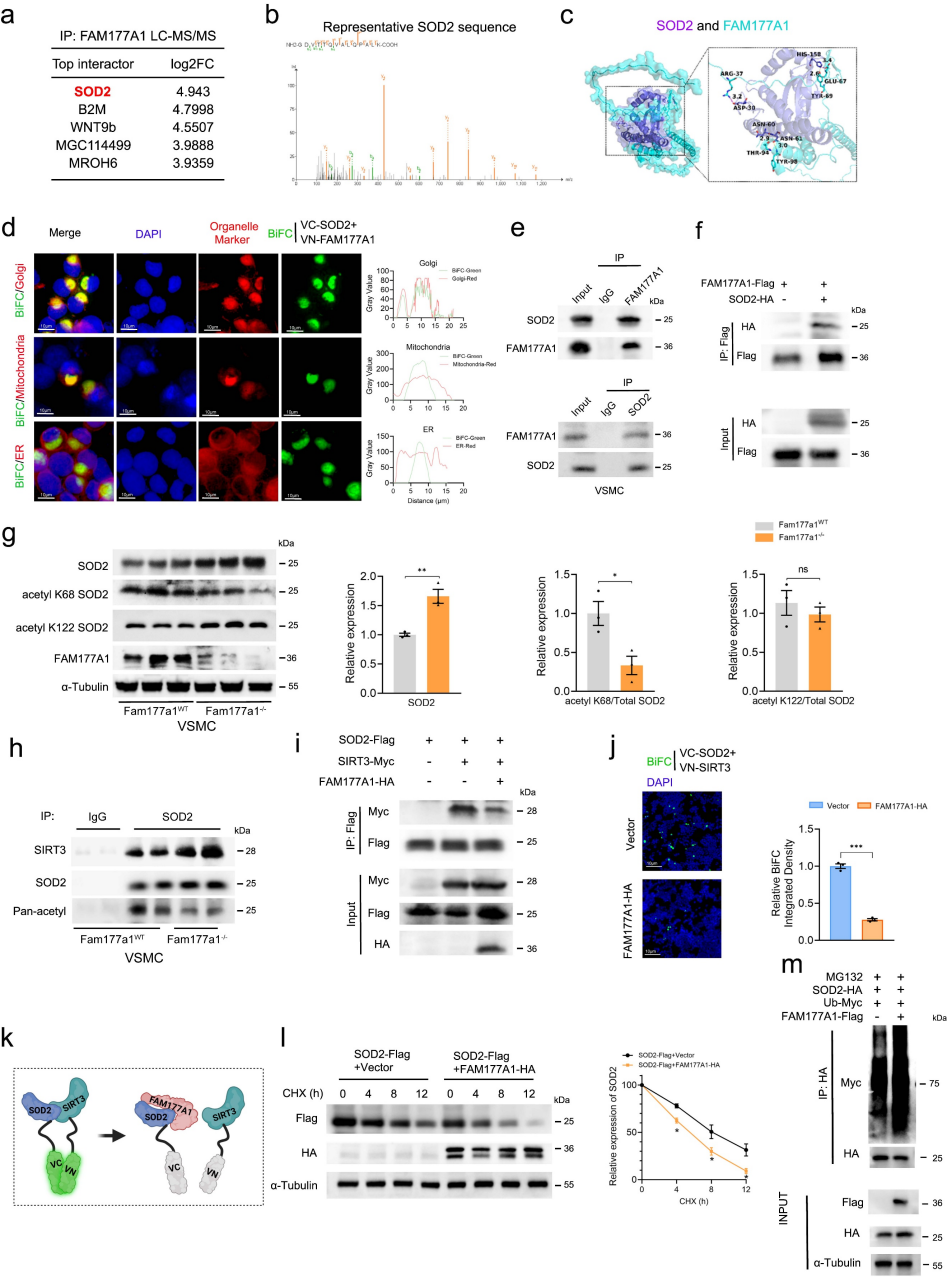
** FAM177A1 interacted with SOD2 by competing with SIRT3. a-b.** Top interactors of FAM177A1 identified by Liquid chromatography-tandem mass spectrometry (LC-MS/MS) (**a**) and the peptide fragments of SOD2 (**b**). **c.** Schematic illustration of molecular docking to predict the binding of SOD2 (purple) and FAM177A1 (blue). **d**. Representative immunofluorescence images of BiFC (green) and organelle marker(red) (58K for Golgi, Mitotracker for mitochondria, Calnexin for ER). **Right**, the co-localization analysis chart. **e**. The interaction of FAM177A1 and SOD2 were determined by co-IP assays in VSMCs. **f**. HEK293T cells were transfected with plasmids expressing SOD2 and FAM177A1 and subjected to co-IP assay. **g**. Representative Western blotting and quantification of SOD2, acetyl K68 SOD2, acetyl K122, FAM177A1 protein levels in *Fam177a1*^WT^ and *Fam177a1*^-/-^ VSMCs (n = 3). α-Tubulin was used for control. Relative expression level of SOD2, acetyl K68/total SOD2, acetyl K122/total SOD2 were shown on the analysis chart (**Right**). **h**. The interactions of SOD2 and SIRT3 were determined by co-IP assays in *Fam177a1*^WT^ and *Fam177a1*^-/-^ VSMCs.** i**. Representative Western blotting of the interactions of SOD2 and SIRT3 in HEK293T cells.** j**. Representative immunofluorescence images of BiFC (green) assays validating effects of FAM177A1 overexpression on the interactions of SOD2 and SIRT3 (scale bar: 10μm). **k**. Schematic illustration of FAM177A1 interacted with SOD2 by competing with SIRT3. **l**. Representative Western blotting and quantification of SOD2 in HEK293T cells transfected with plasmids expressing SOD2 and FAM177A1 followed by CHX (25μM) treatment at different time points (n = 3).** m.** Representative Western blotting of SOD2 polyubiquitination assays with FAM177A1overexpression in HEK293T cells**.** All Data are presented as means ± SEM. P-values are calculated using the Student's t-test (unpaired) analysis in **g, j** and **l**. *p <0.05, **p<0.01, ***p<0.001. LC-MS/MS, liquid chromatography-tandem mass spectrometry; BiFC, bimolecular fluorescence complementation.

To determine how FAM177A1 regulates mitochondrial function, we performed immunoprecipitation mass spectrometry (IP-MS/MS) in VSMCs to identify its binding partners. Intriguingly, SOD2, a key mitochondrial antioxidant enzyme, emerged as the top interactor (Fig. 6a-b). Protein-protein docking of AlphaFold-predicted structures (SOD2: slate; FAM177A1: cyan) identified multiple hydrogen-bond-forming residue pairs, including FAM177A1-Ser435 and SOD2-Ser387, with a strong binding score of -640 (Fig. 6c). As FAM177A1 was poorly characterized, we examined its subcellular localization in VSMCs. Immunofluorescence revealed tight perinuclear aggregates asymmetrically positioned near the nucleus, consistent with its reported localization as a membrane-associated protein predominantly in the Golgi complex^27^. FAM177A1 co-localized perfectly with the Golgi apparatus and partially with mitochondria, but was absent from the cytoskeleton and endoplasmic reticulum (Fig. S5a). Bimolecular fluorescence complementation (BiFC) in HEK293 cells expressing SOD2-VC155 and FAM177A1-VN173 confirmed cytoplasmic interaction (Fig. S5b). This signal co-localized with Golgi and mitochondria (Fig. 6d), consistent with known SOD2/FAM177A1 distribution. Endogenous and exogenous co-immunoprecipitation validated their interaction in VSMCs and HEK293T cells, respectively (Fig. 6e-f). Truncation mapping revealed FAM177A1 binds SOD2^100-180^, while SOD2 binds FAM177A1^64-236^ (Fig. S5c), aligning with AlphaFold docking predictions.

### FAM177A1 disrupts SIRT3-SOD2 binding to exacerbate PDGF-BB induced SOD2 suppression

In light of the above mechanistic findings, we hypothesized that FAM177A1 would lead to mitochondrial oxidative stress and impaired function by disrupting SIRT3-SOD2 binding. SOD2 scavenges superoxide produced by respiratory chain enzymes and counters mitochondrial ROS (mtROS), playing a critical role in protecting against vascular remodeling-associated mitochondrial dysfunction, including vascular stiffness, calcification, and atherosclerosis^26^. Notably, SOD2 mRNA and protein levels initially increased upon early PDGF-BB stimulation but subsequently declined, suggesting a cellular self-protective response to oxidative stress. With prolonged stimulation, SOD2 peaked at 12h and decreased progressively by 24h (Fig. S6a-b). *Fam177a1*^-/-^ VSMCs resisted PDGF-BB-induced SOD2 suppression, exhibiting higher SOD2 protein levels with reduced K68 acetylation versus *Fam177a1*^WT^ controls (Fig. 6g).

Since SIRT3, the primary deacetylase targeting SOD2, preserves VSMCs from oxidative stress by maintaining SOD2 activity^26^, ^50^, we investigated whether FAM177A1 regulates SOD2 acetylation via SIRT3. However, neither overexpression nor knockout of *Fam177a1* altered SIRT3 expression (Fig. S6c-d). We therefore hypothesized that FAM177A1 disrupts SIRT3-SOD2 binding. Endogenous co-IP in PDGF-BB-stimulated VSMCs (48h) confirmed reduced SIRT3-SOD2 interaction (Fig. S6e). Consistently, *Fam177a1*^-/-^ VSMCs showed enhanced SIRT3-SOD2 binding and decreased acetylation level of SOD2 versus* Fam177a1^WT^* (Fig. 6h). Exogenous co-IP and BiFC in HEK293 cells further demonstrated that FAM177A1 overexpression diminished SIRT3-SOD2 interaction (Fig. 6i-k). In addition to SOD2, SIRT3 has other substrates, such as IDH2 and LCAD. To investigate whether the interaction and acetylation between SIRT3 and SOD2 are specific, we examined the acetylation levels of two other substrates, IDH2 and LCAD, in both *Fam177a1*^WT^and *Fam177a1*^-/-^ VSMCs. The Western blot results showed that, unlike the findings for SOD2, the acetylation levels of IDH2 and LCAD remained unchanged in *Fam177a1*^WT^and *Fam177a1*^-/-^ VSMCs, and no significant differences were observed in the protein expression levels of IDH2 and LCAD in the input lanes. This may be related to the specific binding of FAM177A1 to SOD2, which inhibits the interaction between SIRT3 and SOD2 (Fig. S6f-g). FAM177A1 overexpression also decreased SOD2 stability and promoted ubiquitin-mediated degradation (Fig. 6l-m). Using a deacetylation-mimic SOD2 K68R mutant, we found that blocking K68 acetylation reversed FAM177A1-induced SOD2 reduction and ubiquitination (Fig. S6h-i). Collectively, the binding of FAM177A1 with SOD2 impedes the SIRT3-SOD2 interaction, promoting SOD2 K68 hyperacetylation, which subsequently compromises SOD2 protein stability and leads to its degradation.

### Suppression of SOD2 reversed protective effect of *Fam177a1*^-/-^ on VSMCs phenotypic switch in balloon injury-induced neointimal hyperplasia or PDGF-BB stimulation models

**Figure 7:**
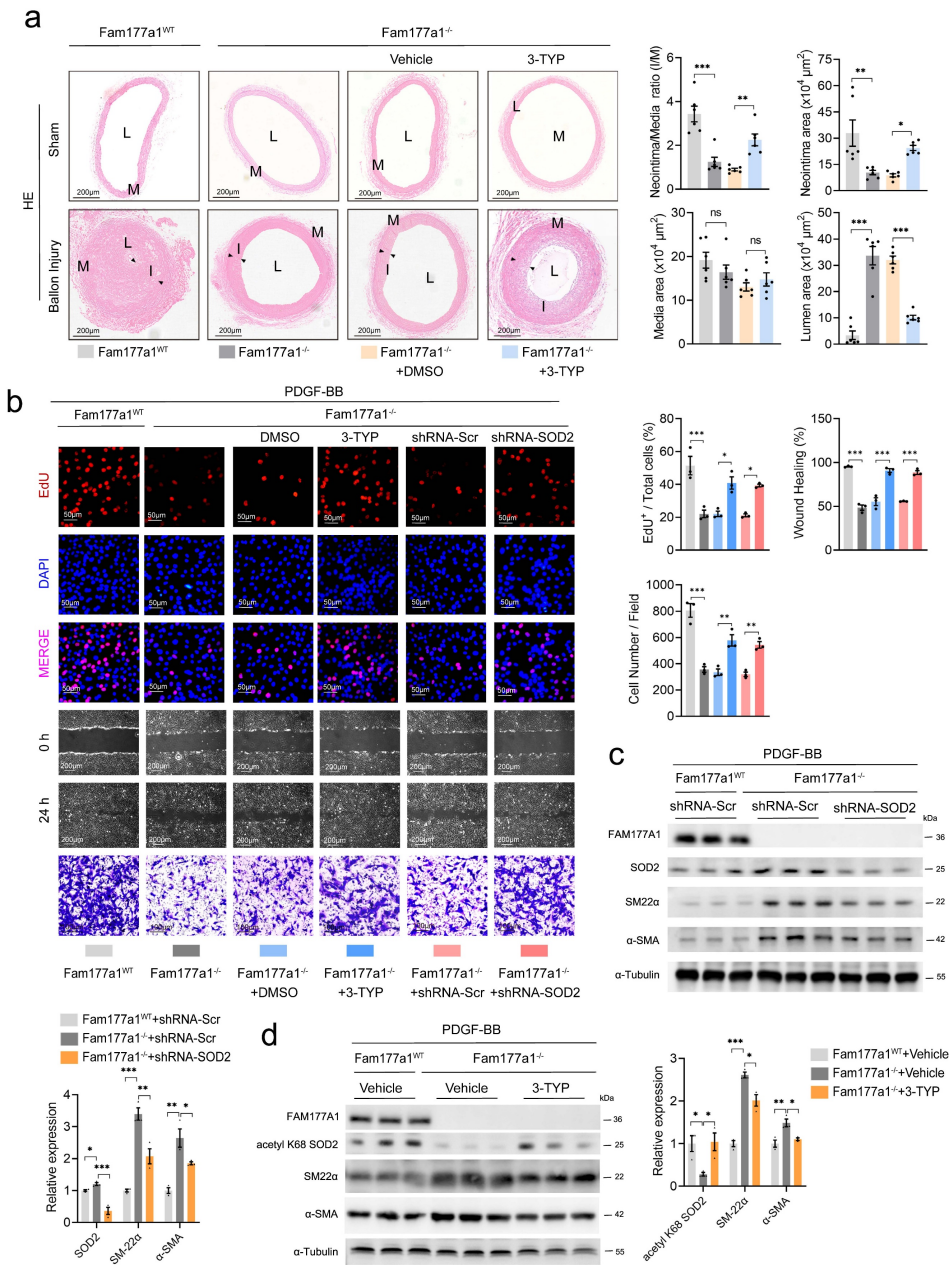
** SOD2 interference abrogated the effects of* Fam177a1* deletion. a.** Representative cross sections of H&E and Masson in male *Fam177a1*^WT^ and *Fam177a1*^-/-^ rats carotid arteries post ballon injury 28 days intraperitoneally injected of 3-TYP (50mg/kg/d) (scale bar:200μm). **Right**, quantitative analysis of the neointima/media ratio(I/M), neointima areas, media areas and lumen areas (n = 6).** b**. The effects of *Sod2* knockdown and inhibition on cell proliferation (EdU assays, scale bar: 50μm) and cell migration (wound healing assays, scale bar: 200μm and Transwell assays, scale bar: 100μm) in *Fam177a1*^-/-^ VSMCs. The EdU^+^/total cells ratio, wound healing rate and cell numbers per field were analyzed in the statistical chart (n = 3). **c**. Representative Western blotting and quantification of FAM177A1, SM22α, αSMA, SOD2 expression in *Fam177a1*^WT^ and* Fam177a1*^-/-^ VSMCs treated with Ad-shRNA-Scr or Ad-shRNA-SOD2. α-Tubulin was used for control. Data were presented as relative fold change to Ad-shRNA-Scr+*Fam177a1*^WT^ group. **d**. Representative Western blotting and quantification of FAM177A1, SM22α, αSMA, acetyl K68 SOD2 expression in *Fam177a1*^WT^ and* Fam177a1*^-/-^ VSMCs treated with DMSO or 3-TYP (30μm). α-Tubulin was used for control. Data were presented as relative fold change toVehicle+*Fam177a1*^WT^ group. All Data are presented as means ± SEM. P-values are calculated using the one-way ANOVA with a post hoc test of Tukey's analysis in **a-d**. *p <0.05, **p<0.01, ***p<0.001. 3-TYP, 3-(1H-1,2,3-triazol-4-yl) pyridine, selective SIRT3 inhibitor.

Given FAM177A1's role as a critical regulator of the SIRT3-SOD2 axis, we inhibited SOD2 expression and activity to determine whether it functions as a primary downstream effector. Ballon-catheter injury was performed in *Fam177a1*^WT^ and *Fam177a1*^-/-^ rats. After ballon injury, *Fam177a1*^-/-^ rats were randomly grouped and the 3-TYP group rats were injected intraperitoneally of 3-TYP (50mg/kg/d), a selective SIRT3 inhibitor once a day to inhibit SIRT3-SOD2 activity. Twenty-eight days post-injury, histological analysis (H&E and Masson staining) revealed that 3-TYP significantly reversed the protective effect of *Fam177a1*^-/-^ against vascular intimal hyperplasia (Fig. 7a). The 3-TYP group showed increased neointimal area and I/M ratio with reduced lumen area versus vehicle in *Fam177a1*^-/-^ rats (Fig. 7a).

**Figure 8:**
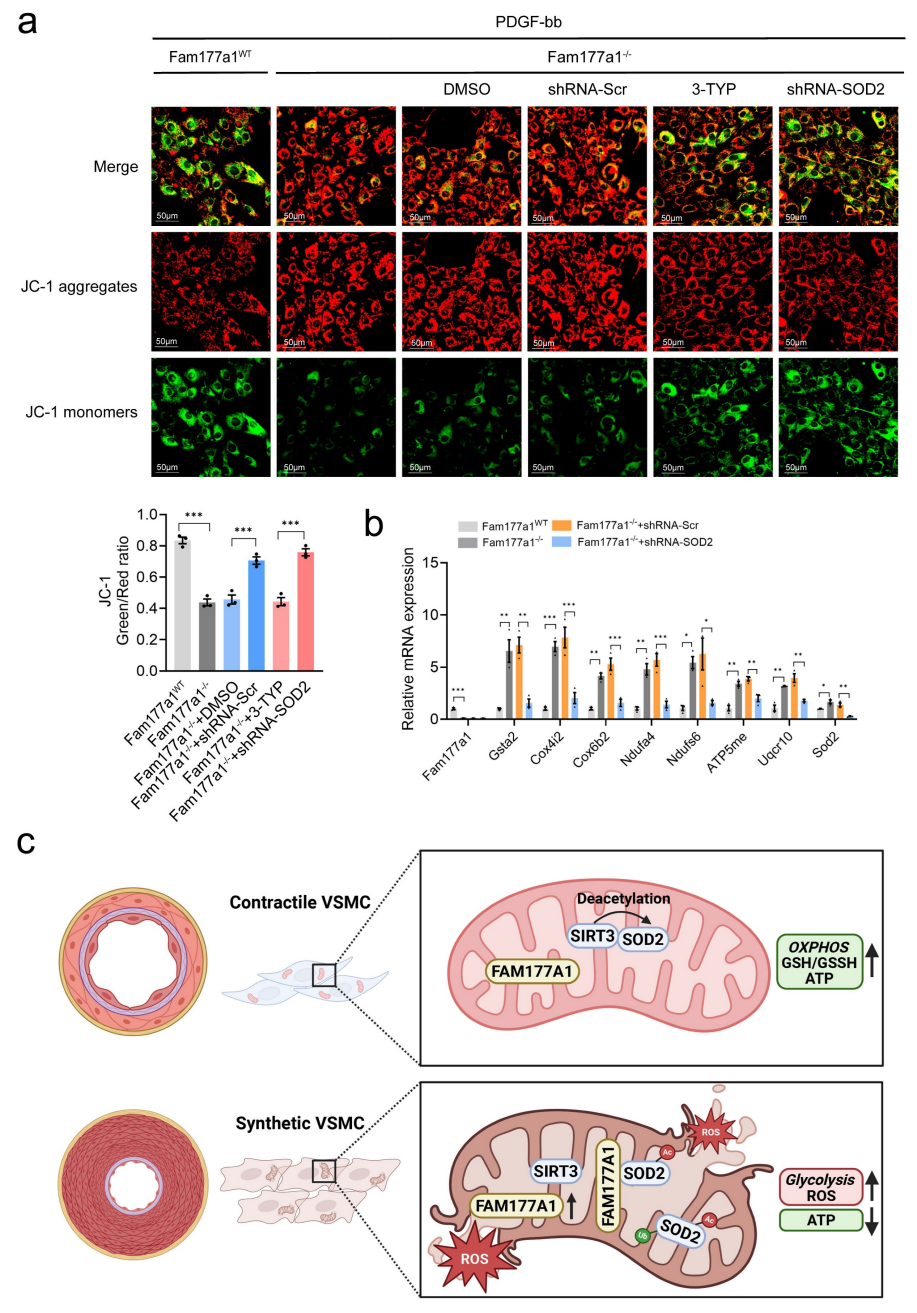
** SOD2 interference abrogated the protective effects of *Fam177a1* deletion on maintain mitochondria function. a.** Representative immunofluorescence images and quantification of JC-1 staining showing membrane potential by confocal fluorescence microscopy of *Fam177a1*^WT^ and *Fam177a1*^-/-^ VSMCs after PDGF-BB (20ng/ml, 48h) induction, with different treatment of DMSO, 3-TYP, Ad-shRNA-Scr and Ad-shRNA-SOD2 (n = 3) (scale bar: 50 μm). b. qPCR validation of mitochondria and oxidative phosphorylation related genes in* Fam177a1*^WT^ and* Fam177a1*^-/-^ VSMCs after PDGF-BB (20ng/ml, 48h) induction, with different treatment of DMSO, 3-TYP, Ad-shRNA-Scr and Ad-shRNA-SOD2 (n = 3). 18S was used for control. All Data are presented as means ± SEM. P-values are calculated using the one-way ANOVA with a post hoc test of Tukey's analysis in **a** and **b**. *p <0.05, **p<0.01, ***p<0.001.

In primary VSMCs, besides pharmacological inhibition of SOD2, we knocked down *Sod2* via shRNA (5′-GGAUUGAUGUGUGGGAGCACGCUUA-3′). Both approaches reversed *Fam177a1*^-/-^-mediated suppression of proliferation/migration in EdU, wound healing, and Transwell assays (Fig. 7b). Western blot confirmed that 3-TYP increased SOD2 K68 acetylation, and shRNA*-*mediated* Sod2* knockdown in *Fam177a1*^-/-^ VSMCs (Fig. 7c-d). Moreover, both interventions reversed *Fam177a1*^-/-^-mediated changes in contractile protein profiles (Fig. 7c-d). Measurement of ΔΨm using JC-1 probe and qPCR analysis of key mitochondrial oxidative phosphorylation genes revealed that both treatments abrogated the protective effects of *Fam177a1* deficiency on mitochondrial function and oxidative phosphorylation (Fig. 8a-b).

In summary, our study reveals for the first time that FAM177A1 regulates both the expression and activity of the critical antioxidant protein SOD2 in mitochondria. This mechanism modulates mitochondrial oxidative phosphorylation in VSMCs, enhancing cellular antioxidant capacity and ROS generation under vascular remodeling. Upon stimulation, increased FAM177A1 expression disrupts the SIRT3-SOD2 interaction, elevating SOD2 K68 acetylation while decreasing SOD2 activity and stability. These changes collectively impair mitochondrial function, increase mtROS production, and promote VSMC phenotypic switching and pathological vascular remodeling (Fig. 8c). Given FAM177A1's secretory nature in humans, our findings highlight its potential as a therapeutic target for vascular remodeling.

## Discussion

Cardiovascular diseases remain a major global health burden, exacerbated by aging populations and metabolic disorders. VSMCs, which are essential for maintaining vascular integrity^51^, undergo detrimental phenotypic switching in pathological conditions such as atherosclerosis and restenosis. This switching is characterized by abnormal proliferation, migration, and loss of contractile function^52^, ^53^. In recent years, there has been a growing body of research on the metabolic reprogramming of VSMCs regulating their phenotypic changes, which plays a role in various vascular diseases^54^. Mitochondria precisely regulate glucose metabolism, lipid metabolism, and amino acid metabolism through the tricarboxylic acid cycle and oxidative phosphorylation, which is crucial for maintaining metabolic homeostasis. Recent single-cell RNA sequencing studies have shown that widespread mitochondrial dysfunction occurs in various types of aortic cells and is one of the hallmarks of aortic aneurysm^55^. An increasing number of studies have also reported that genes involved in the regulation of VSMC phenotypes may influence their phenotypes through mitochondrial function^56^. Additionally, dysregulated mitochondrial homeostasis and increased glycolysis pathways lead to the involvement of glycolytic metabolites, such as lactate, in VSMC regulation. It has been reported that increased lactate can mediate elevated PARP expression and PARylation in VSMCs^48^, and may also be involved in epigenetic modifications, such as participating in histone lactylation at the H3K18^57^, ^58^ or H3K9^59^ sites, thereby regulating VSMC or endothelial cell functions and contributing to processes like calcification, atherosclerosis and aortic aneurysm. While mitochondrial dysfunction in VSMCs has been implicated in these processes, the specific molecular regulators connecting mitochondrial homeostasis to phenotypic plasticity remain incompletely understood.

VSMCs are essential for maintaining vascular integrity but undergo detrimental phenotypic switching under pathological conditions, characterized by aberrant proliferation, migration, and loss of contractile function^60^. In this study, we identify FAM177A1 as a novel and critical regulator of pathological vascular remodeling through its disruption of mitochondrial redox-metabolic homeostasis. FAM177A1 is an evolutionarily conserved protein that is widely expressed in mammals and widely reported localized to the Golgi apparatus, belonging to the FAM177 protein family. Although functional studies on FAM177A1 remain limited, recent advances in molecular biology have gradually uncovered its involvement in multiple biological processes. Ko et al. elucidated a sophisticated regulatory paradigm in mouse embryonal carcinoma cells, wherein FAM177A1 expression is precisely modulated by the neural-specific microRNA miR-124a^61^. Their work established that FAM177A1 exhibited marked upregulation during proliferative phases while being potently suppressed during neuronal differentiation. Recent RNA sequencing in human fibroblast also demonstrated of FAM177A1 deficiency showed negative regulation of cell proliferation^28^. Liao et al. identified FAM177A1 as a negative regulator of IL-1β-induced signaling by inhibiting TRAF6-Ubc13 interaction^29^. Recent studies have primarily focused on the functional characterization of FAM177A1 in the nervous system. Patients with homozygous frameshift mutations in FAM177A1 typically present with early-onset neurodevelopmental abnormalities followed by neurodegenerative progression, including deteriorating motor function, refractory progressive epilepsy, arthritis/neuroinflammation, along with macrocephaly, muscular tone disorders, gait disturbance, global developmental delay, distinctive facial dysmorphism, and mild obesity^62^. In summary, although its functional roles remain incompletely characterized, emerging evidence implicates FAM177A1 in several biological processes—including cell proliferation, lipid metabolism, and innate immune response —all of which are closely associated with vascular pathological remodeling. This prompted us to investigate the role of FAM177A1 in VSMC phenotypic regulation.

We first observed significant upregulation of FAM177A1 in phenotypically modulated VSMCs within atherosclerotic lesions, following mechanical injury, or upon PDGF-BB stimulation. It has been reported that VSMCs can switch to myofibroblast-like phenotypes^63^ which occurs within human AS plaque^64^. Myofibroblasts display a phenotype intermediate between fibroblasts and vascular smooth muscle cells (VSMCs), typically observed in pathological contexts. They are responsible for the production extracellular matrix components such as type I and III collagens and fibronectin—as well as secreting inflammatory cytokines^65^. Published single-cell RNA sequencing data (GSE174098) from rat carotid arteries after balloon injury demonstrated a dynamic increase in *Fam177a1* expression during vascular stenosis, with particular enrichment in phenotypic switching subpopulations-Fibroblasts. Elevated FAM177A1 in Fibroblast group marked contractile VSMCs transition to a fibroblast-like state, contributing to neointima formation.

In addition, we validated the upstream transcriptional regulation of FAM177A1 using dual-luciferase reporter assays. An intriguing and unexpected finding from our promoter analysis was that, despite the well-established role of ELK1 as an upstream transcriptional activator of the c-JUN gene itself, overexpression of ELK1 did not impair the PDGF-BB-induced activation of the *Fam177a1* promoter. This suggests a distinct and specific regulatory logic for the* Fam177a1* gene. It appears that while the upstream PDGF-BB signal may utilize the ERK-ELK1 axis to induce c-Jun expression, the* Fam177a1* promoter itself is not directly controlled by ELK1. Instead, it is selectively and directly responsive to the downstream transcription factor complex c-Jun/AP-1, which converges with the independently activated STAT3 pathway. This places FAM177A1 in a specific regulatory niche, likely as a secondary or late-response gene that integrates signals from both the AP-1 and STAT3 pathways to execute a precise transcriptional program in response to PDGF-BB, rather than being part of the immediate-early gene set directly governed by ELK1/SRF. This specificity in promoter architecture and transcription factor requirement underscores the layered complexity of the transcriptional network activated by PDGF-BB in VSMCs.

Importantly, elevated circulating levels of FAM177A1 were detected in atherosclerosis patients, highlighting its potential as a clinical biomarker. Given the presence of a secretory isoform in humans, serum FAM177A1 may serve as a non-invasive indicator of vascular remodeling progression. Consistent with this, both gain- and loss-of-function experiments, in vivo and in vitro, demonstrated that FAM177A1 drives the transition of VSMCs from a contractile to a synthetic phenotype.

Unexpectedly, immunoprecipitation mass spectrometry identified SOD2 as a major interacting partner of FAM177A1. This finding was unexpected given SOD2's well-established mitochondrial localization, which contrasts with the canonical Golgi association of FAM177A1. Subsequent validation using immunofluorescence and BiFC confirmed the dual localization of FAM177A1 to both the Golgi apparatus and mitochondria, and demonstrated strong co-localization of the FAM177A1-SOD2 complex within mitochondria. The Golgi apparatus serves as a pivotal organelle in cellular metabolism, involved in modifying, sorting, and packaging macromolecules for secretion or intracellular use^66^. It has also recently been implicated in the regulation of mitochondrial respiration, division, and dynamics^67^-^69^. While Golgi-derived vesicles containing PI(4)P have been recognized as important mediators of mitochondrial and ER-mitochondria crosstalk^69^, protein-mediated inter-organelle interactions remain poorly understood. Thus, the subcellular distribution and regulatory mechanisms governing FAM177A1 localization clearly warrant further investigation. In addition, since the Golgi-related biological system is highly responsive to mitochondrial ROS/RNS-mediated oxidative stress and participates in damage repair^70^, the current study and future investigations could significantly advance our understanding of these mechanisms.

While SIRT3-SOD2 signaling is recognized as a protective pathway against vascular oxidative stress, our data further reveal FAM177A1 acts as a unique molecular disruptor by physically interfering with SIRT3-SOD2 binding. This interference dysregulates the critical acetylation/deacetylation dynamics controlling SOD2 enzyme activity. We further observed that FAM177A1 promotes SOD2 polyubiquitination and degradation. Given FAM177A1's known interaction with the E3 ubiquitin ligase TRAF6^29^, and considering other uncharacterized ligases may participate, the precise mechanisms of FAM177A1-mediated SOD2 degradation warrant further investigation. The FAM177A1-SIRT3-SOD2 axis profoundly impacts mitochondrial gene expression and function. FAM177A1 deficiency upregulated oxidative phosphorylation genes and enhanced mitochondrial respiration, while suppressing glycolytic flux. This metabolic reprogramming likely occurs through two complementary mechanisms: direct restoration of mitochondrial function, and activation of mito-nuclear communication pathways that trigger nuclear gene expression changes—creating a positive feedback loop reinforcing mitochondrial health and contractile phenotype maintenance.

Mitochondrial damage impairs the respiratory chain in VSMCs, leading to excessive ROS production and reduced ATP levels, which are also associated with VSMC proliferation, migration, and apoptosis^71^. Here, we demonstrate that pathological upregulation of FAM177A1 in vascular smooth muscle cells drives phenotypic switching by directly binding SOD2 and inhibiting its interaction with the deacetylase SIRT3. This disruption elevates SOD2 K68 acetylation, reduces SOD2 stability and activity, and ultimately causes mitochondrial dysfunction characterized by ROS accumulation, loss of membrane potential (ΔΨm), and impaired mitochondrial oxidative phosphorylation and morphology. Crucially, genetic ablation of FAM177A1 enhances SIRT3-SOD2 binding, restoring SOD2 function and promoting a contractile VSMC phenotype through coordinated improvement of redox balance and bioenergetic efficiency. Consistent protective effects across multiple models confirm that targeting FAM177A1 suppresses neointimal hyperplasia and atherosclerotic plaque progression.

Therapeutically, strategies to inhibit FAM177A1, such as monoclonal antibodies or small-molecule disruptors of FAM177A1-SOD2 binding, show significant promise. Rescue experiments using SIRT3 inhibition or SOD2 knockdown confirmed the SIRT3-SOD2 axis as the dominant downstream effector pathway. Importantly, future therapeutic development should prioritize VSMC-specific targeting to mitigate potential off-organ effects, given FAM177A1's roles in neurological and immune functions. Key unanswered questions include the differential contributions of FAM177A1 isoforms (secreted vs. membrane-bound) in vascular pathology, potential crosstalk with other SIRT3 substrates (e.g., IDH2, LCAD)^72^, and identification of upstream regulators controlling FAM177A1 expression during VSMC phenotypic switching.

## Conclusion

In summary, this work identifies FAM177A1 as a critical orchestrator of vascular remodeling through mitochondrial dysregulation. By disrupting SIRT3-SOD2 signaling, it initiates a self-amplifying cycle of oxidative damage and metabolic dysfunction that drives pathological VSMC phenotypic switching. Therapeutic targeting of this axis offers a dual-mechanism strategy—simultaneously improving redox balance and bioenergetic efficiency—to restore vascular homeostasis. These findings position FAM177A1 as a compelling diagnostic biomarker and therapeutic target for cardiovascular diseases characterized by vascular remodeling.

## Supplementary Material

Supplementary methods, figures and tables.
