# Supplementary material for: FAM177A1 disrupts SIRT3-SOD2 signaling to drive mitochondrial dysfunction-mediated VSMC phenotypic switching in vascular remodeling
Source: Int J Biol Sci. 2026 Feb 26;22(6):3108–26. doi: 10.7150/ijbs.128409 (PMC13050452; doi:10.7150/ijbs.128409)
Supplement: Supplementary file 1 — Supplementary methods, figures and tables. [file ijbsv22p3108s1.pdf]

Supplementary Materials

Method

Public datasets and patient samples

To examine the expression pattern of FAM177A1 and mitochondria associated genes in normal and diseased arteries, we used the R software (version 4.1.0; R Foundation for Statistical Computing, Vienna, Austria) for public data analysis. GSE174098 were obtained from Gene Expression Omnibus. For the collection of serum from healthy individuals and patients with atherosclerosis, we strictly followed the patient selection criteria. Blood samples were obtained from eligible participants among those receiving physical examinations in the Department of Geriatrics at Union Hospital, Huazhong University of Science and Technology. Informed consent was obtained from all participants, and the collection procedures were approved by the Ethics Committee of Huazhong University of Science and Technology, in accordance with the principles of the Declaration of Helsinki. After centrifugation, serum was collected for subsequent ELISA and other tests. Detailed characteristics of the physical examination cohort are presented in **Supplementary Table S2**.

Serum ELISA assay

The ELISA kit was custom-developed by HYCEZMBIO® (HYCM12405, China), utilizing antibodies from Atlas (HPA055440, Sweden). The assays were strictly performed according to the manufacturer's protocols. Measure the OD values of test samples and standards at 450 nm, then determine the FAM77A1 concentration by comparing the sample absorbance against the standard curve. Each sample was tested in triplicate, and the average value was calculated.

Dual-Luciferase Reporter Assay

Fam177a1 promoter sequence was amplified by PCR and cloned into the multiple cloning site of the firefly luciferase reporter vector (pGL3.0-Basic). A *Renilla* luciferase expression vector (pRL-TK) was used as an internal control for normalization of transfection efficiency. A7r5 cells line were cultured in DMEM supplemented with 10% fetal bovine serum at 37°C in a 5% CO_2 humidified incubator. For the assay, cells were seeded in 24-well plates and grown to approximately 70–80% confluence. Each well was co-transfected with:10ng pRL-TK, 500ng pGL3.0-Basic or pGL3.0-*Fam177a1*-promoter and 500ng Vector (Vec) /STAT3/c-JUN/ELK1 plasmids. Transient transfection was performed using PEI. After48 hours of transfection, cells were washed with PBS and lysed with Lysis Buffer according to the Dual Luciferase Reporter Gene Assay Kit (Beyotime, RG027). Luciferase and *Renilla* activity was measured using the Dual-Luciferase® Reporter Assay System (Promega) according to the manufacturer's instructions. The firefly luciferase activity for each sample was normalized to the *Renilla* luciferase activity.

Cell culture

For rat primary VSMCs, male Sprague–Dawley rats (150–200 g, n = 2–3) were anesthetized by intraperitoneal injection of 0.5% sodium pentobarbital and euthanized. The thoracic aorta was isolated, carefully stripped of perivascular adipose tissue, and incubated in type II collagenase solution (2 mg/ml, Worthington, USA) for 15 min at 37°C. The adventitia was carefully removed, and the aorta was washed with PBS solution and cultured overnight in DMEM supplemented with 10% FBS (Gibco) at 37°C in a 5% CO_2 incubator. The next day, the aorta was minced into 1 mm^2 fragments and digested in a mixture of type II collagenase (2 mg/ml, Worthington) and elastase (1 mg/ml, Sangon Biotech, Shanghai, China) for 90 min at 37°C in a 5% CO_2 incubator. The digested tissue was dissociated into a single-cell suspension, centrifuged, and resuspended in DMEM with 10% FBS for plating. RVSMCs were studied at passages 3-8 or until senescence was reached. T/G HA-VSMC (Procell, China) passages 3-5 were used for phenotypic experiments and cultured in HA-VSMC-specific medium (Procell, China). HEK293A and HEK293T (Procell, China) were cultured in DMEM with 10% FBS (F103, Vazyme). For some experiments, RVSMCs and HEK293T cells were treated with PDGF-BB (100-14B, Peprotech), SIRT3 inhibitor 3-TYP (HY-108331, MCE), cycloheximide (HY-12320, MCE) and MG132(HY-13259, MCE).

Analysis of mRNA expression

Total RNA of tissues and cells were extracted by RNAiso Plus (9109, Takara) and reverse transcribed (R323, Vazyme). qPCR was performed with ChamQ Universal SYBR qPCR Master Mix (Q711, Vazyme) and an ABI 7500 Real-Time PCR system (Thermo Fisher Scientific). All genes relative fold changes were normalized to 18S and calculated with $2^{-\Delta\Delta CT}$. Primers for q-PCR were listed in Supplementary Table S1. RNA-seq was performed (Bioyigene, Wuhan, China) using extracted RNA from passage 3 wild-type and *Fam177a1* knockout rat VSMCs. Subsequent bioinformatics analyses including differential expression, Gene Ontology enrichment, and GSEA were conducted by R package.

Protein extraction and Western blot analysis

For the cell protein extraction, the treated cells were scraped off using RIPA lysis buffer （Servicebio） mixed with protease and phosphatase inhibitors(PHOSS-RO; COEDTAF-RO, Roche, Sigma Aldrich), followed by ultrasonication and centrifugation to collect the supernatant. For vascular tissue, Minute™ Total Protein Extraction Kit for Blood Vessels (SA-03-BV, Invent) was used to extract vascular tissue protein according to the manufacturer's instructions. Protein concentration was determined using the BCA assay (23227, Thermo Scientific™, USA). Proteins were separated by sodium dodecyl sulfate polyacrylamide gel electrophoresis (SDS-PAGE) and transferred onto a PVDF membrane (IPVH00010, Millipore). The membrane was blocked with 5% non-fat milk at room temperature for 1 hour, followed by overnight incubation with primary antibody targeting FAM177A1 (HPA055440, Atlas, 1:1000 dilution) , α-SMA (67735-1-Ig , Proteintech, 1:1000 dilution), SM22α (10493-1-AP, Proteintec, 1:1000 dilution), CNN1 (13938-1-AP, Proteintech, 1:1000 dilution), Cyclin D1 (26939-1-AP, Proteintech , 1:1000 dilution), GAPDH (10494-1-AP, Proteintech, 1:1000 dilution), α-Tubulin (14555-1-AP, Proteintech, 1:1000 dilution), SMMHC (21404-1-AP, Proteintech, 1:1000 dilution), PCNA (10205-1-AP,

Proteintech, 1:1000 dilution), MMP9 (10375-2-AP, Proteintech, 1:1000 dilution), SOD2 (24127-1-AP, Proteintech, 1:1000 dilution), HA (RA1004, Vazyme), Flag (F1804, Sigma), MYC (60003-2-Ig, Proteintech, 1:1000 dilution), SOD2/MnSOD (acetyl K68) (ab137037, Abcam, 1:1000 dilution), SOD2/MnSOD (acetyl K122) (ab214675, Abcam, 1:1000 dilution), IDH2(15932-1-AP, Proteintech, 1:1000 dilution), LCAD(17526-1-AP, Proteintech, 1:1000 dilution), SIRT3 (10099-1-AP, Proteintech, 1:1000 dilution), Pan Acetylation (66289-1-Ig, Proteintech, 1:1000 dilution) on a shaker at 4°C. The next day, after HRP-conjugated secondary antibody (SA00001-1 or SA00001-2, Proteintech, 1:10000 dilution) incubation, the target band was visualized using ECL substrate (Servicebio) and a UviTech imaging system.

Immunofluorescence assay

Following deparaffinization, rehydration, and antigen retrieval, vascular tissue sections were blocked with 5% goat serum (Servicebio) for 1 hour, incubated with primary antibody targeting FAM177A1 (HPA055440, Atlas, 1:100 dilution), α-SMA (Proteintech, 67735-1-Ig, 1:250 dilution), CD68 (28058-1-AP, Proteintech, 1:100 dilution) at 4°C overnight in a humid chamber. Next day, washed with PBST, and then incubated with fluorescent secondary antibody (Alexa Fluor 488- and Alexa Fluor 555-conjugated,4409 and 4412, CST, USA) for 2 hours at room temperature protected from light, followed by DAPI (G1012, Servicebio) nuclear staining for 30 minutes. For cell staining, samples were fixed with 4% paraformaldehyde for 15 min at room temperature, permeabilized with 0.3% Triton X-100 for 10 minutes, blocked with 1% BSA for 30 minutes, and incubated with primary antibody. In addition, the following were required: 58K for Golgi (ab27043, Abcam, 1:100 dilution), Calnexin for Endoplasmic Reticulum (ER) (sc-46669, Santa Cruz, 1:50 dilution)) at 4°C overnight, followed by fluorescent secondary antibody (the antibody information as previously described) incubation for 2 hours at room temperature in the dark and DAPI nuclear staining for 30 minutes. JC-1 and MitoTracker from Thermo Fisher Scientific (Thermo, NY, USA) were used for fluorescence confocal microscopy imaging. For phalloidin staining, the samples were directly stained with phalloidin (RM02835, Abclonal, Wuhan, China) for 30 minutes. Fluorescent images were acquired using confocal microscopy (Nikon, Japan), and mean fluorescence intensity with co-localization analysis was performed using ImageJ.

Flow Cytometry

Cell cycle detection was performed according to the instructions of the cell cycle detection kit (C1052, Beyotime, China). Reactive oxygen species (ROS) was detected with the ROS detection kit (S0033, Beyotime, China). Data analysis was performed with FlowJo.

Cell proliferation assays

CCK-8 and EdU were performed to assess cell proliferation. WT and *Fam177a1*$^{-/-}$ rat VSMCs were seeded at 1,000 cells /well in a 96-well plate. After infection Ad-*Fam177a1*, cells were serum-starved overnight in DMEM without FBS, followed by stimulation with PDGF-BB (20 ng/mL) for 24 h. The CCK-8 (C0038, Beyotime) and EdU (C0075, Beyotime) assays were then performed according to the

manufacturers' protocols.

Cell Migration assays

Cell migration ability was assessed using Transwell and wound healing assays. For the Transwell assay, Ad-*Fam177a1* treated RVSMCs were digested, resuspended in serum-free DMEM, and seeded into the upper chamber. The lower chamber was filled with 500 μL of DMEM containing 10% FBS. PDGF-BB was added to the upper chamber according to experimental groups. After overnight migration, the medium in the chambers was removed, and non-migrated cells on the upper side were gently wiped away. The migrated cells in the lower chamber were stained with crystal violet (G1014, Servicebio) and imaged under an Olympus inverted microscope. For the wound healing assay, pre-treated rat VSMCs were seeded in 6-well plates. A scratch was made using a pipette tip, followed by PDGF-BB stimulation. After 24 hours, cell migration into the wound area was photographed using an Olympus inverted microscope. All experiments statistical analysis was performed using ImageJ.

Plasmids Construction and Bimolecular fluorescence complementation (BiFC) assays

The plasmids of FAM177A1, SOD2, SIRT3 and mutant forms were purchased from Gene Universal (Anhui, China) and MiaolingBio (Wuhan, China). The adenovirus and SM22α-promoter-shRNA-*Fam177a1*-AAV were constructed and validated by Hanbio Tech (Shanghai, China) and Gene Universal (Anhui, China). For BiFC assays, SOD2 and SIRT3 were cloned into VC155 vector, FAM177A1 and SIRT3 were cloned into VN173 vector respectively. VN173 and VC155 plasmids carrying the respective target genes were transfected into HEK293T cells, followed by fluorescence microscopy imaging at 36 h post-transfection.

Co-immunoprecipitation (Co-IP), Liquid chromatography-tandem mass spectrometry

Co-IP samples were lysed with IP lysis buffer (20mM Tris-HCl (pH 7.5), 5% glycerol, 1mM EDTA, 150mM NaCl, 1mM $MgCl_2$, 1% NP-40, H_2O to the final volume to 500 mL) containing protease and phosphatase inhibitors (PHOSS-RO; COEDTAF-RO, Roche, Sigma Aldrich) according to the co-IP protocol. IgG or indicated primary antibodies were added to the protein extraction with rotation in 4°C overnight. Next day, protein A/G Magnetic Beads (B23201, Selleck, USA) was added to the protein-antibody mixture and incubated at 4°C with rotation for 2 hours. Subsequently, the beads were washed three times with lysis buffer and three times with wash buffer (20mM Tris-HCl (pH 7.5), 5% glycerol, 1mM EDTA, 150mM NaCl, 1mM $MgCl_2$, H_2O to the final volume to 500 mL). After thorough removal of the liquid, the beads were stored at -80°C. Finally, the samples were shipped on dry ice to the company (SpecAlly, Wuhan, China) for LC-MS/MS analysis. For Co-IP assays, the immunoprecipitated complex and total lysates were boiled for western blotting. Next day, the HRP-conjugated secondary antibody incubation was performed, while the remaining steps followed the same procedure as described above. Secondary anti-bodies: HRP-conjugated Goat anti-Rabbit IgG (SA00001-2, Proteintech); HRP-conjugated Goat anti-Mouse IgG (SA00001-1, Proteintech); anti-rabbit IgG for IP (HRP) (RA1008; Vazyme); anti-mouse IgG for IP (HRP) (RA1009; Vazyme).

ATP concentration measurement

The ATP levels in WT and *Fam177a1*$^{-/-}$ rat VSMCs were measured using an ATP assay kit (S0027, Beyotime). Rat VSMCs were seeded in 6-well plates, treated with lysis buffer, and centrifuged to collect the supernatant. According to the kit instructions, ATP standard solutions at varying concentrations were prepared. The test samples and ATP standards were added to assay tubes, and fluorescence intensity was measured using a microplate reader. The ATP concentration in the samples were calculated based on the standard curve.

GSH/GSSG measurement

The GSH/GSSG levels in WT and *Fam177a1*$^{-/-}$ rat VSMCs were measured using an GSH and GSSG assay kit (S0027, Beyotime, China). The procedure was strictly performed according to the manufacturer's instructions. The GSH concentration and GSH/GSSG ratio were calculated based on the standard curve.

Seahorse assays

OCR (Oxygen Consumption Rate) and ECAR (Extracellular Acidification Rate) of rat VSMCs were measured using the Seahorse XFe24 Analyzer (Seahorse Biosciences, Agilent, USA) according to the manufacturer's instructions. WT and *Fam177a1*$^{-/-}$ VSMCs were seeded at 1.5×10^5 cells/well in Seahorse 24-well microplates with 10% FBS DMEM medium and cultured overnight. The medium was then replaced with pre-warmed Seahorse XF Base Medium (pre-mixed with 1 mM pyruvate, 2 mM L-Glutamine, 10 mM Glucose). For ECAR measurement, only 2 mM L-Glutamine was additionally supplemented to the Seahorse XF base medium. Initiate the Mitochondrial Stress Test program to measure the baseline OCR of the samples, followed by sequential OCR measurements after the addition of the following inhibitors: 2 μM oligomycin for ATP synthase inhibitor, 1 μM FCCP for mitochondrial uncoupler and 1 μM rotenone/antimycin A for complex I/III inhibitors. For ECAR measurement, the following inhibitors are sequentially added: 10 mM Glucose, 1 μM oligomycin, 50 mM 2-deoxyglucose. Results were normalized to total protein concentration determined by BCA assay (23227, Thermo Scientific™) and analyzed using Wave software (Agilent).

Mitochondria DNA copy number measurement

Isolate genomic DNA from target cell samples strictly following the manufacturer's instructions of the DNA extraction kit. After determining the DNA concentration, the mitochondrial D-LOOP content was detected by q-PCR. Mitochondrial DNA-specific primer sequences were detailed in the **Table S1**.

Electron microscopy

Electron microscopy was performed according to the sample submission guidelines of Servicebio. Briefly, rat VSMCs were fixed in electron microscope-specific fixative solution at room temperature for

5 minutes. VSMCs were gently scraped off using a cell scraper. The cells were then centrifuged 1000 rpm for 5 minutes and resuspended using a specialized fixative solution before being sent to Servicebio for subsequent processing and imaging.

Statistical analysis

All data were analyzed and figures were plotted using GraphPad Prism 8 or R software. Values are presented as the mean ± SEM. The data were first tested for normality using the Shapiro-Wilk test. For normally distributed data, comparisons between two groups were analyzed by standard Student's t test, while comparisons among multiple groups were performed using one-/ two-way ANOVA with a post hoc test of Tukey's analysis. All statistical analyses were conducted with at least three independent experiments, and a $P < 0.05$ was considered statistically significant.

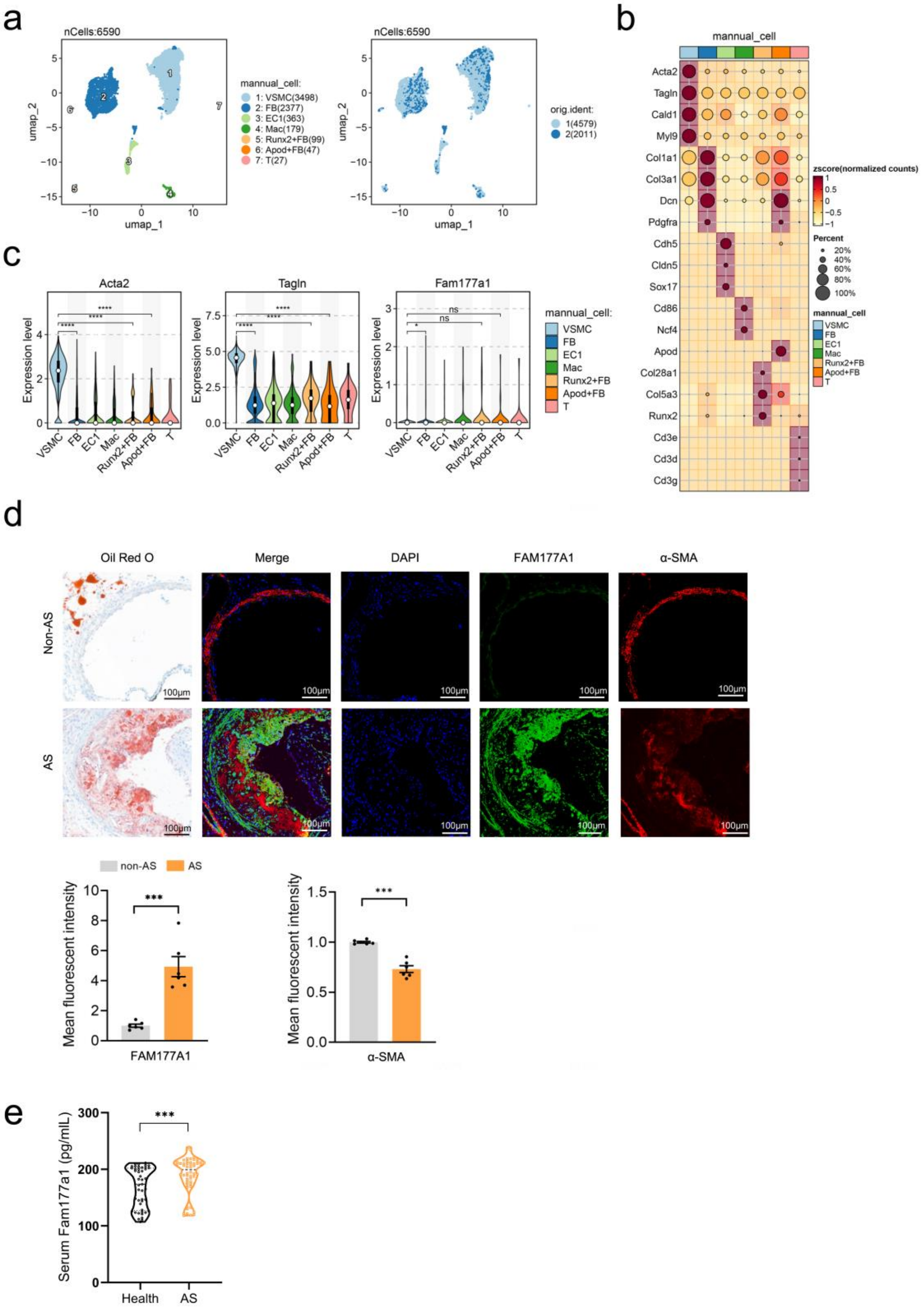

Figure S1. FAM177A1 expression correlates with pathological vascular remodeling. a. Cell population clustering revealed by UMAP dimensionality reduction projection (GSE174098). **b**. Heatmap visualization of cluster-specific marker genes. **c**. Violin plots showing *Acta2, Tagln,* and *Fam177a1* expression in cell clusters (*vs*. VSMC controls). **d**. Representative Oil Red O staining and immunofluorescence images and quantification of α-SMA (red) and FAM177A1 (green) in aortic root of *Apoe*$^{-/-}$ +HFD (AS) mice and *Apoe*$^{-/-}$+NCD (non-AS) mice (n = 6). DAPI was used for nuclear staining (blue). Scale bar: 100μm.

Statistical analysis of FAM177A1 and αSMA mean fluorescence intensity (total intensity/area) were shown below (both normalized to non-AS group). **e**. ELISA detection of FAM177A1 levels in serum from healthy controls (n=54) and AS patients (n=45). All Data are presented as means ± SEM. P-values are calculated using the Student's t-test (unpaired) analysis in **d**, the one-way ANOVA with a post hoc test of Tukey's analysis in data **c** and Mann-Whitney U test in **e.** **p* <0.05, ***p*<0.01, ****p*<0.001. Acta2 (α-SMA), α-smooth muscle actin; Tagln (SM22α), transgelin; AS, atherosclerosis; HFD, high fat diet; NCD, normal chow diet.

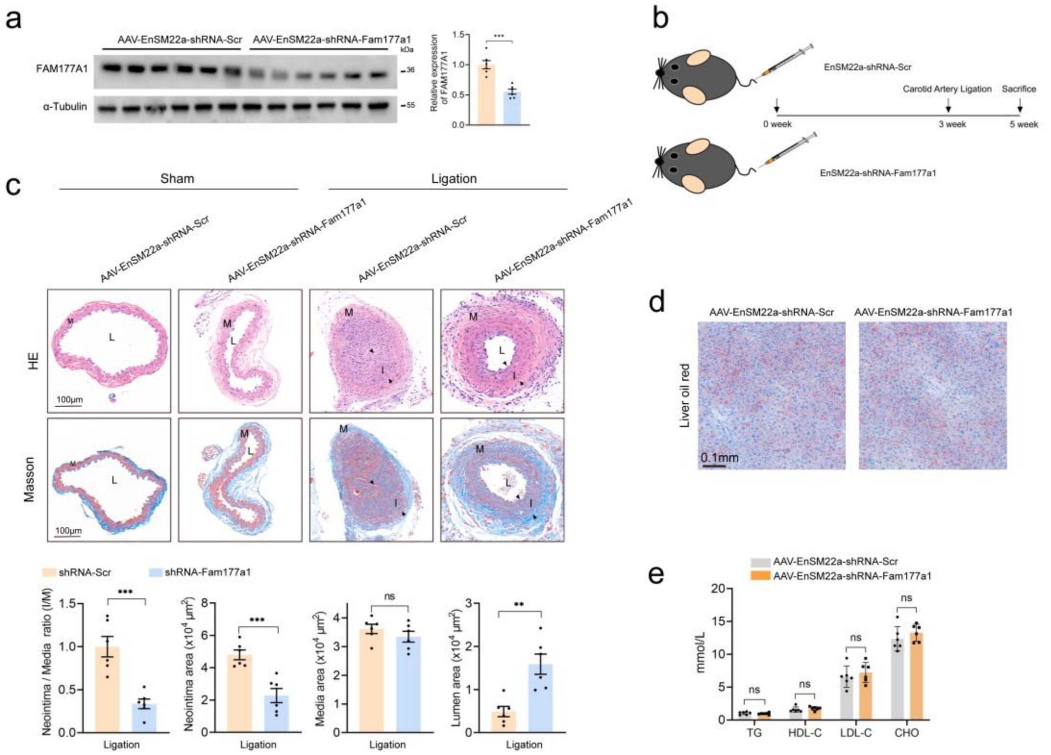

Figure S2. *Fam177a1* specific knockdown in VSMCs alleviated vascular neointima hyperplasia *in vivo*. a. Representative Western blotting and quantification of *Fam177a1* knockdown efficiency in aortas of male mice 3 weeks after injection with AAV-EnSM22α-shRNA-Scramble or AAV-EnSM22α-shRNA-*Fam177a1* via the tail vein (n = 6). α-Tubulin was used for control. Data were presented as relative fold change to AAV-EnSM22α-shRNA-Scramble group. **b**. Experimental scheme. Wildtype C57BL/6J mice were injected with AAV-EnSM22α-shRNA-Scramble or AAV-EnSM22α-shRNA-*Fam177a1* via the tail vein, followed by carotid arteries ligation or sham operation. Bilateral carotid arteries were collected for further analysis at 2 weeks. **c**. **Up**, representative cross sections of H&E and Masson in male mice carotid arteries injected with AAV-EnSM22α-shRNA-Scramble or AAV-EnSM22α-shRNA-*Fam177a1* after carotid arteries ligation or sham operation. Scale bar: 100μm. **Down**, quantitative analysis of the neointima/media ratio (I/M), neointima areas, media areas and lumen areas in HE staining from mice as indicated (n = 6). **d**. Representative cross-sectional images of Oil Red O-stained liver sections from AAV-EnSM22α-shRNA-Scramble and AAV-EnSM22α-shRNA-*Fam177a1* transfected *Apoe*$^{-/-}$+HFD (AS) mice (scale bar: 0.1mm). **e**. Quantitative analysis of serum TG, HDL-C, LDL-C and CHO content from AAV-EnSM22α-shRNA-Scramble and AAV-EnSM22α-shRNA-Fam177a1 transfected *Apoe*$^{-/-}$+HFD (AS) mice (n = 6 per group). All Data are presented as means ± SEM. P-values are calculated using the Student's t-test (unpaired) in **a, c-e**. **p* <0.05, ***p*<0.01, ****p*<0.001. TG, triglyceride; HDL-C, high-density lipoprotein cholesterol; LDL-C, low-

Density lipoprotein cholesterol; CHO, cholesterol.

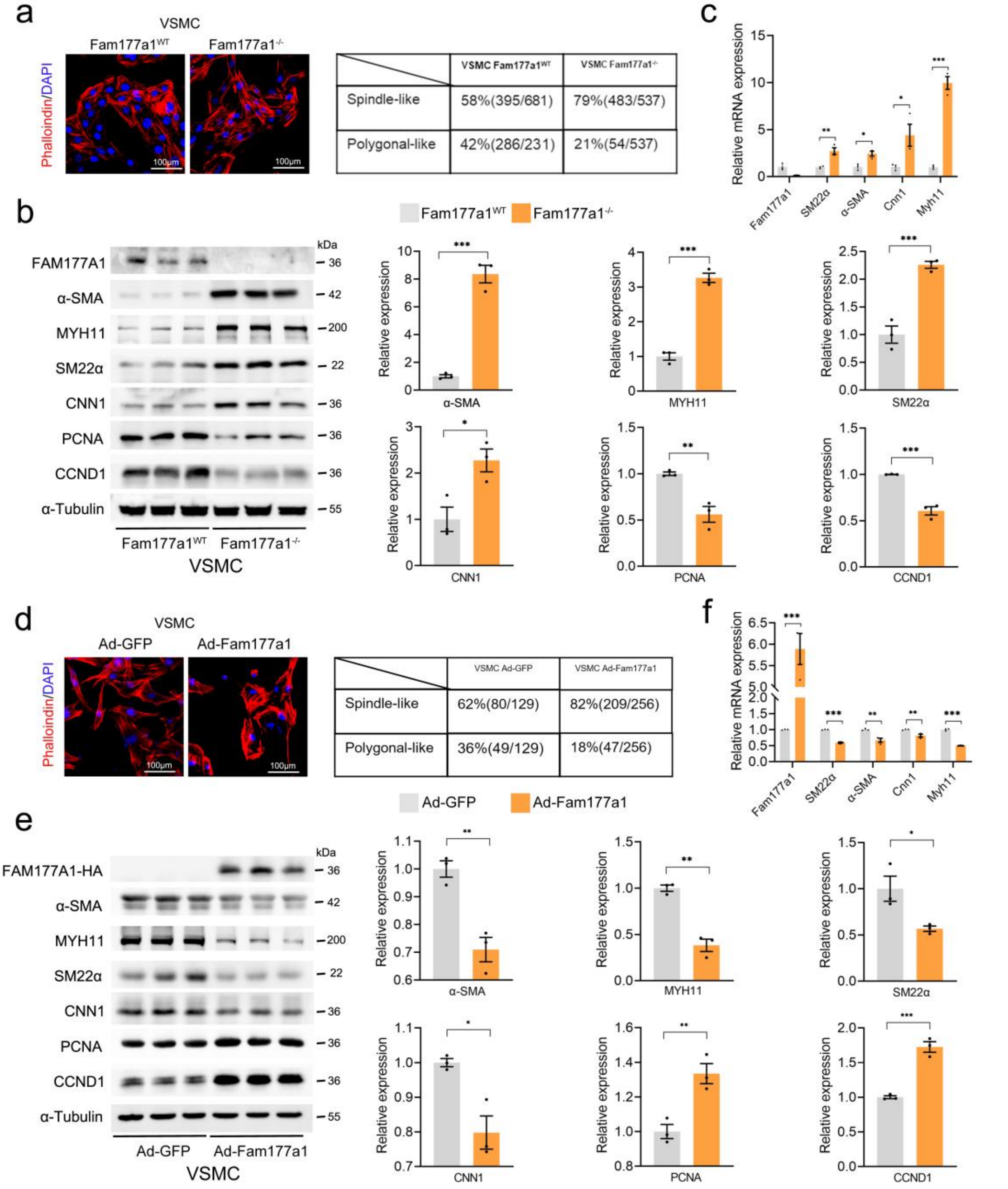

Figure S3. FAM177A1 modulated VSMCs phenotypic switch *in vitro*. a. Representative immunofluorescence images of phalloindin (red) in *Fam177a1*^{WT} and *Fam177a1*$^{-/-}$ rat VSMCs. DAPI was used for nuclear staining (blue). Scale bar: 100μm. **b**. Representative Western blotting and quantification of FAM177A1, SM22α, αSMA, MYH11, CNN1, PCNA and CCND1 expression in *Fam177a1*^{WT} and *Fam177a1*$^{-/-}$ rat VSMCs. α-Tubulin was used for control. Data were presented as relative fold change to *Fam177a1*^{WT} group. **c**. qPCR analysis of the relative mRNA level of *Fam177a1, SM22α, αSMA, Cnn1* and *Myh11* in rat VSMCs (n = 3). 18S was used for control. **d.** Representative immunofluorescence images of phalloindin (red) in Ad-GFP and Ad-

FAM177A1 transfected VSMCs. DAPI was used for nuclear staining (blue). Scale bar: 100μm. **e**. Representative Western blotting and quantification of FAM177A1, SM22α, αSMA, MYH11, CNN1, PCNA and CCND1 expression in VSMCs as indicated. α-Tubulin was used for control. Data were presented as relative fold change to Ad-GFP group. **f**. qPCR analysis of the relative mRNA level of F*am177a1, SM22α, αSMA, Cnn1* and *Myh11* in VSMCs as indicated (n = 3). 18S was used for control. All Data are presented as means ± SEM. P-values are calculated using Student's t-test (unpaired) analysis in **b, c, e** and **f**. *p <0.05, **p<0.01, ***p<0.001.

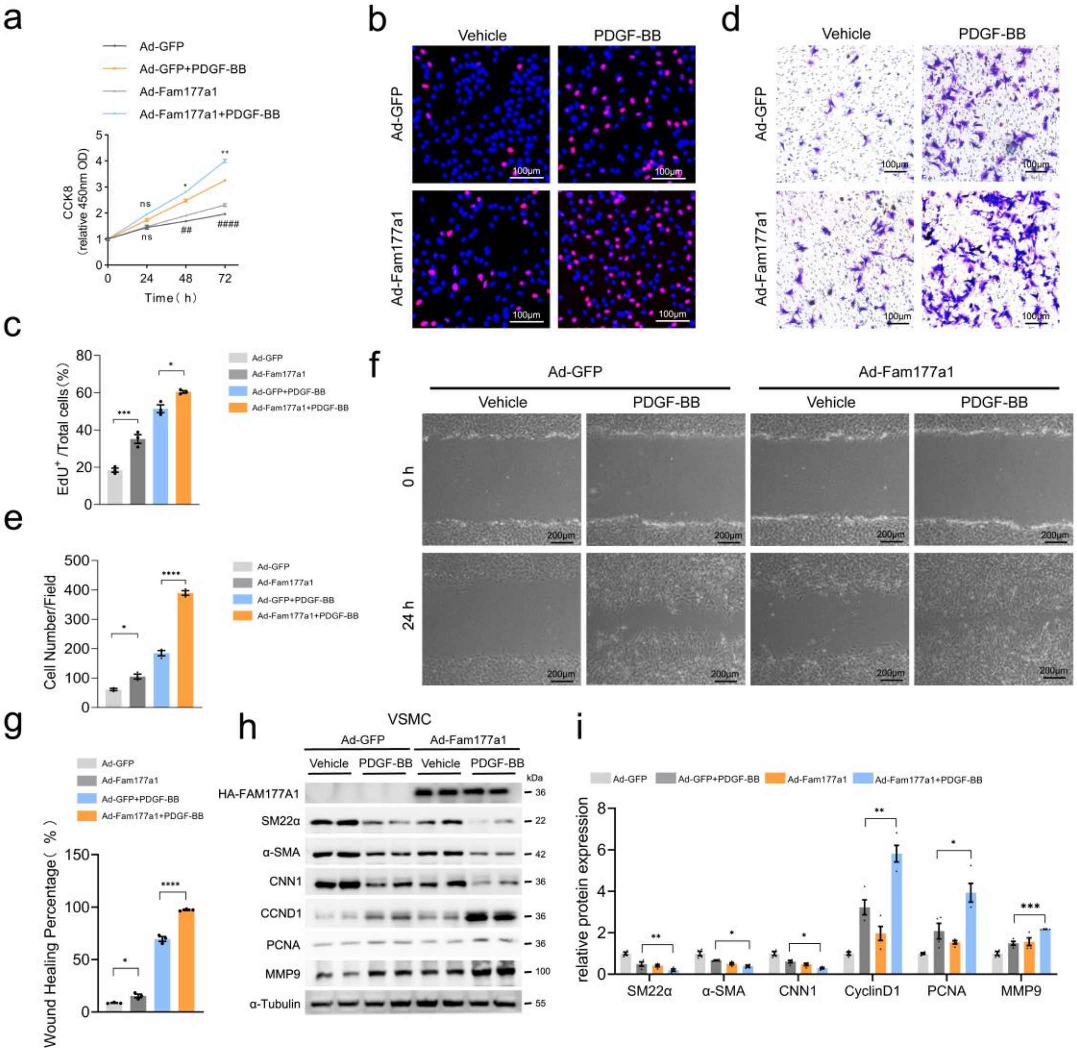

Figure S4. FAM177A1 inhibited VSMCs contractile phenotype *in vitro*. a, b, c, d, e. Ad-GFP and Ad-FAM177A1 transfected rat VSMCs were stimulated with vehicle or PDGF-bb (20ng/ml). CCK8 assays (**a**), EdU staining (**b**) were used to examine cell proliferation (**b**, scale bar: 100μm). Cell migration was examined using Transwell (**d**) and wound healing assays (**f**) (**d**, scale bar: 100μm; **f**, scale bar: 200μm). EdU^{+}/total ratio (**c**), cell count per field (**e**) and wound healing rate (**g**) were evaluated in the statistical chart (n = 3 per group). **h-i**. Representative Western blotting and quantification of HA-Fam177a1, SM22α, αSMA, CNN1, PCNA, MMP9 expression in Ad-GFP and Ad-FAM177A1 transfected rat VSMCs with or with our PDGF-BB (20ng/ml, 48h) treatment.

α-Tubulin was used for control. Data were presented as relative fold change to control group. All Data are presented as means ± SEM. P-values are calculated using the one-way ANOVA with a post hoc test of Tukey's analysis in data **a**, **c**, **e**, **g** & **i**. *p <0.05, **p<0.01, ***p<0.001.

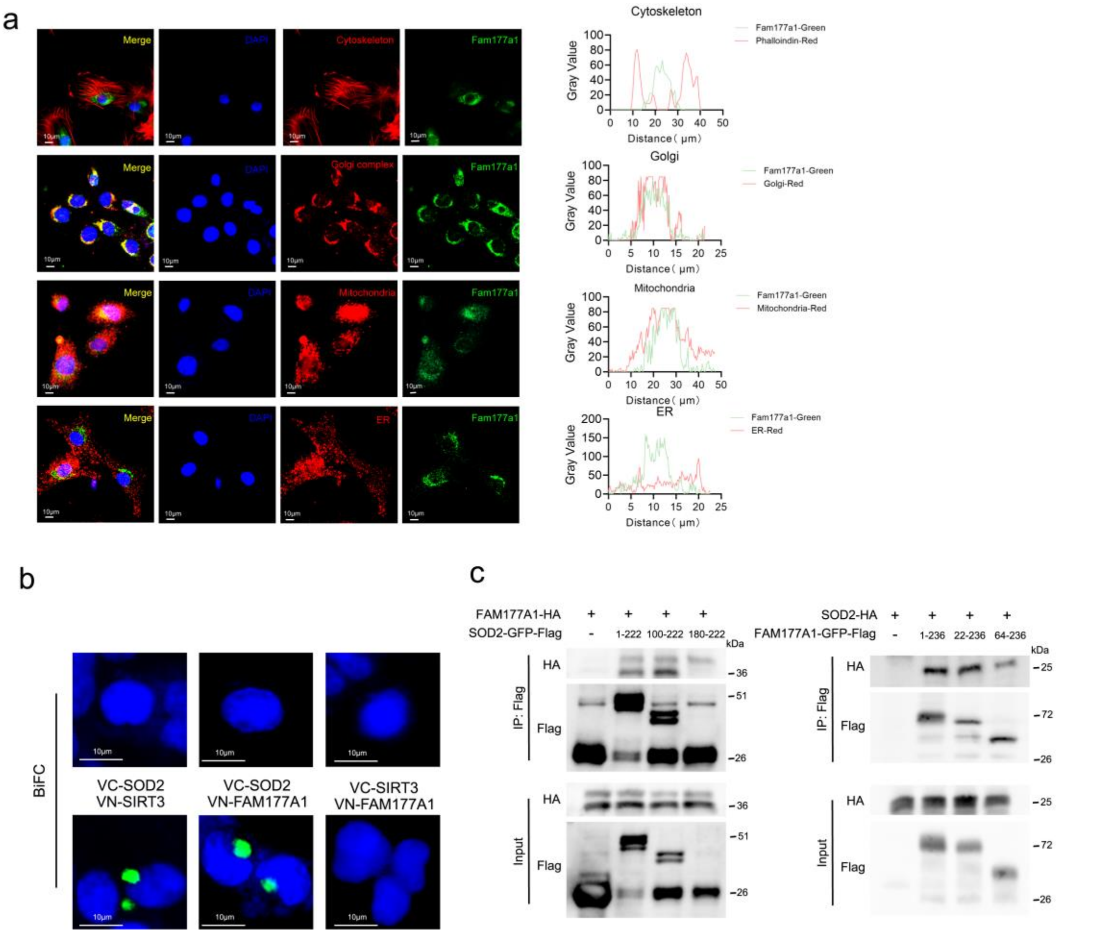

Figure S5. FAM177A1 localized to the Golgi apparatus and mitochondria and interacted with SOD2. a. Representative immunofluorescence images of FAM177A1 (green) and different cell organelle markers (red) (Scale bar: 10μm). **b**. HEK293T cells were transfected with SOD2, SIRT3 and FAM177A1 cloned into VC155 or VN173 constructs for BiFC (green) assays. DAPI was used for nuclear staining. **c**. HEK293T cells were transfected with plasmids expressing different domains of FAM177A1 and SOD2 subjected to co-IP.

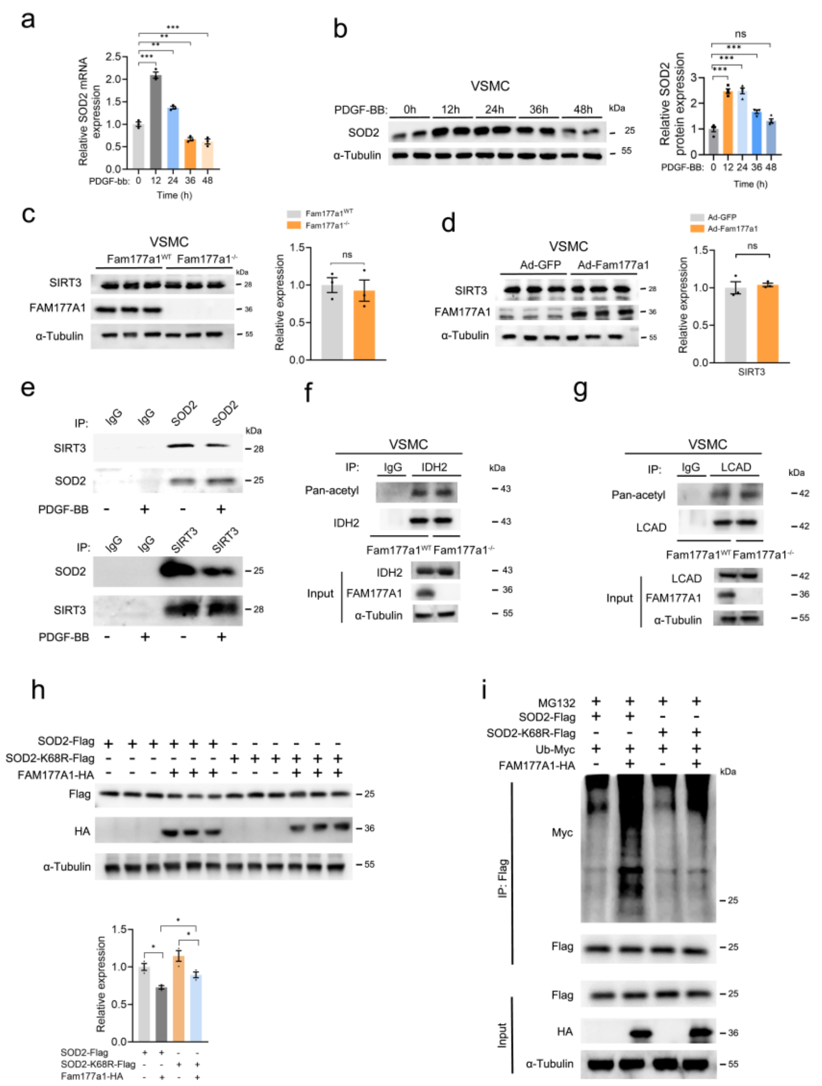

Figure S6. FAM177A1 modulated SOD2 expression and activity. a. qPCR analysis of the relative mRNA level of *Sod2* in rat VSMCs upon PDGF-bb (20ng/ml) stimulation at different time intervals (n = 3). 18S was used for control. **b**. Representative Western blotting and quantification of SOD2 expression in rat VSMCs as indicated (n = 3). α-Tubulin was used for control. **c.** Representative Western blotting and quantification of SIRT3 expression in *Fam177a1*^{WT} and *Fam177a1*$^{-/-}$ rat VSMCs (n = 3). α-Tubulin was used for control. **d.** Representative Western blotting and quantification of SIRT3 expression in Ad-GFP and Ad-FAM177A1 transfected VSMCs (n = 3). α-Tubulin was used for control. (n = 3). **e**. The interactions of SOD2 and SIRT3 were determined by co-IP assays in rat VSMCs upon PDGF-bb (20ng/ml) stimulation. **f**. The acetylated level of IDH2 was assessed by co-IP assays in *Fam177a1*^{WT} and *Fam177a1*$^{-/-}$ rat VSMCs. **g**. The acetylated level of LCAD was assessed by co-IP assays in *Fam177a1*^{WT} and *Fam177a1*$^{-/-}$ rat VSMCs. **h**. HEK293T cells were transfected with plasmids expressing SOD2-Flag, SOD2-K68R-Flag and FAM177A1-HA. Representative Western blotting and quantification of SOD2 expression in HEK293T cells to validate the effect of SOD2 acetylation on SOD2 protein levels (n = 3). α-Tubulin was used for control. **i**. Representative Western blotting of polyubiquitination of SOD2 in HEK293T cells to validate the effect of SOD2 acetylation on SOD2 polyubiquitination levels. All Data are presented as means ± SEM. P-values are calculated using the one-way ANOVA with a post hoc test of Tukey's analysis in **a**, **b** and **h**. P-values are calculated using Student's t-test (unpaired) analysis in **c-d**.*p <0.05, **p<0.01, ***p<0.001. IDH2, Isocitrate Dehydrogenase 2; LCAD, Long-Chain Acyl-CoA Dehydrogenase

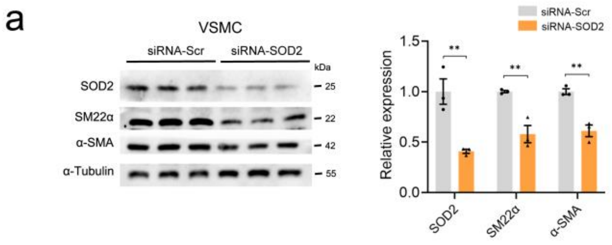

Figure S7. SOD2 deficiency changed the contractile proteins levels in VSMCs. a. Representative Western blotting and quantification of SOD2, SM22α and α-SMA expression in siRNA-Scr and siRNA-SOD2 transfected rat VSMCs. α-Tubulin was used for control. Data were presented as relative fold change to siRNA-Scr group. All Data are presented as means ± SEM. P-values are calculated using Student's t-test (unpaired) analysis in **a** and **b**. *p <0.05, **p<0.01.

Supplementary Table S1. RT-qPCR primers were used in this study.

	Forward sequence	Reverse sequence
Rat		
Acta2	CCTCCAGAACAGATTTGAGAGTAGT	GGGTCAGGGGTGGTTATTGC
Tagln	GACATACTCCAAACCTTTCCACCC	TTCAAAAACTTCTCCACAACCCTC
Cnn1	TTGCCAGCCAGTGACACAAT	GAGAAGGTGGTTGTCTGGGAAT
Myh11	AGGTTGAACCCCACAGTCAC	TCTGAGACCTCTGGCTTCGT
SOD2	GATCTCAGTGCAGAGGCTCG	TCTGGGGAAAGCTAGGGGAA
Sirt3	AGGACACCATGAGCACTGAAAGC	AAGGAGAAGAGGCTGAGGAACAAG
Fam177a1	TGGTTTTGCAGGAGGCTATAC	GTAGATGGCCGAGCGGTACT
Gsta2	GTTGGGGGACCGCTCATT	CCCGGCCATCCTTATAGACG
Cox4i2	TGGAATGCCGGCCAAGCGAC	GCCTCAACCCAGGCATCACCA
Cox6b2	AGCCCTCTTCATGTTCCGAAGTGT	TCATGTCAACACCTGCAGTCCCTT
Ndufa4	GCGCTCTACCGGGAATACC	CAGCCCGATTCAGATGGGG
Ndufs6	TACCCAACCTTGGCTAGACG	GTCCGAGGAGAGAGCTTGC
ATP5me	CCCCGAAGCCTCTACAATGG	CTGAGCTTGGAATCGGACCTT
Uqcr10	GGTCCTCACTTTAATCCTCTATCCAG	CCAACATGCCTCTCTTCATCC

Supplementary Table S2. The basic information of the healthy controls and atherosclerosis patients collected in this study.

Factor	**Normal (n=54)**	**AS (n=45)**	**P-value**
**Sex**			
Female	10	8	> 0.999
Male	44	37	
**Age(years)**	57.0±5.5	57.9±5.1	0.3931
**BMI**	22.93±3.52	23.78±3.45	0.2298
**Blood Glucose (mmol/L)**	4.91±0.42	5.92±0.87	<0.0001
**HDL-cholesterol** (mmol/L)	1.44±0.20	0.95±0.27	<0.0001
**LDL-cholesterol** (mmol/L)	2.51±0.49	2.82±0.62	0.0068
**TG** (mmol/L)	1.42±1.38	1.51±0.96	0.8035
**TC** (mmol/L)	4.55±0.54	4.87±0.70	0.0132
